# Thinking outside the brain: Gut microbiome influence on innate immunity within neurodegenerative disease

**DOI:** 10.1016/j.neurot.2024.e00476

**Published:** 2024-10-31

**Authors:** Andrea R. Merchak, MacKenzie L. Bolen, Malú Gámez Tansey, Kelly B. Menees

**Affiliations:** a Department of Neuroscience, College of Medicine, University of Florida, Gainesville, FL, USA; b Center for Translational Research in Neurodegenerative Disease, College of Medicine, University of Florida, Gainesville, FL, USA; c McKnight Brain Institute, University of Florida, Gainesville, FL, USA; d Aligning Science Across Parkinson's (ASAP) Collaborative Research Network, Chevy Chase, MD, USA; e Norman Fixel Institute for Neurological Diseases, University of Florida, Gainesville, FL, USA

**Keywords:** Microbiome, Innate immunity, Neurodegeneration

## Abstract

The complex network of factors that contribute to neurodegeneration have hampered the discovery of effective preventative measures. While much work has focused on brain-first therapeutics, it is becoming evident that physiological changes outside of the brain are the best target for early interventions. Specifically, myeloid cells, including peripheral macrophages and microglia, are a sensitive population of cells whose activity can directly impact neuronal health. Myeloid cell activity includes cytokine production, migration, debris clearance, and phagocytosis. Environmental measures that can modulate these activities range from toxin exposure to diet. However, one of the most influential mediators of myeloid fitness is the gut microenvironment. Here, we review the current data about the role of myeloid cells in gastrointestinal disorders, Parkinson's disease, dementia, and multiple sclerosis. We then delve into the gut microbiota modulating therapies available and clinical evidence for their use in neurodegeneration. Modulating lifestyle and environmental mediators of inflammation are one of the most promising interventions for neurodegeneration and a systematic and concerted effort to examine these factors in healthy aging is the next frontier.

## Graphical abstract

**Figure:**
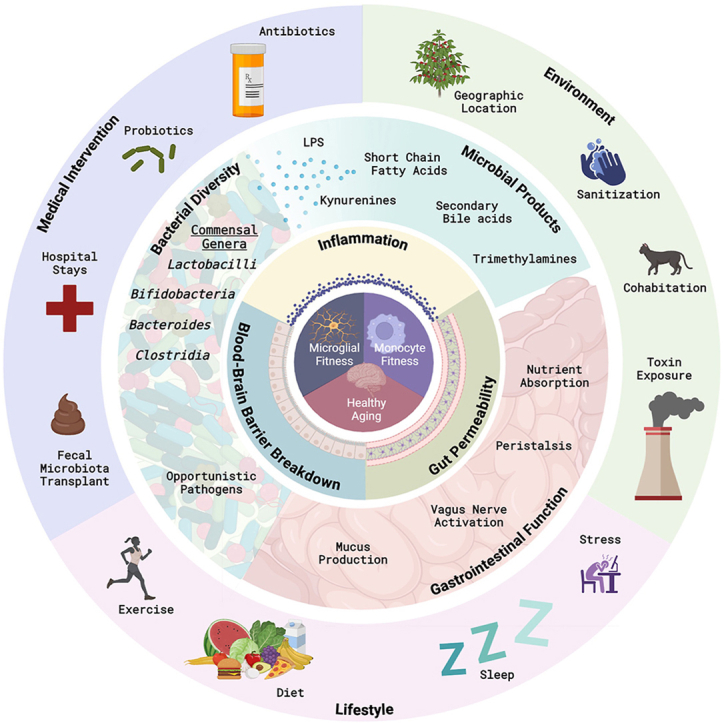

## Introduction

There is rich and complicated communication between the gut, peripheral immune system and central nervous system (CNS). The brain is immune specialized, however, heavily interconnected with peripheral immune dynamics. Peripheral immune cells enter and exit the brain under a variety of conditions and this traffic is part of an important crosstalk between the brain and peripheral organs [1]. Tightly regulated bidirectional communication between brain-resident and peripheral innate immune cells has recently been established to play a critical role in brain health [2].

The gut is the largest immune organ in the body making it a critical space for peripheral immune development, thus influencing brain health [3]. The gut microbiome is a commensal ecosystem comprised of trillions of bacteria, fungi, and viruses that aid in pathogen defense, immune development, and nutrient absorption [4]. Under healthy conditions, the gut microbiota interact with the brain via several mechanisms including microbiota-derived metabolites that enter circulation, by direct communication via the vagus nerve, and through modulation of the immune system. Disruption in the gut microbial communities can lead to gut dysbiosis and potentially contribute to gastrointestinal (GI) dysfunction. This disruption often leads to the breakdown of tight junctions that prevent pathogen infiltration through the gut lining and is termed a “leaky gut”. Leaky gut is an early phenotype observed in several neurodegenerative disorders. The leaky gut leads to an accumulation of inflammatory signals and cells which can exacerbate or induce the onset of neurodegeneration. Conditions which have been shown to disrupt these barriers include environmental exposures or toxicants, lifestyle choices (diet, smoking, alcohol, lack of physical activity), and the process of aging itself [5,6]. The endotoxin hypothesis directly emphasizes this idea where common toxicants such as pesticides are proposed to catalyze gut inflammation, resulting in immune system activation, and associated neurodegenerative risk [7,8]. Therefore, the question arises as to whether these and other external factors that contribute to one's lifetime risk for age-related neurodegenerative diseases exert their actions via modulation of the gut microbiome which then affects the crosstalk between the periphery and the brain.

Millions of dollars have been invested over the past five decades in search of treatments and preventative measures for neurodegenerative diseases. While knowledge of the biology of these disorders has advanced, there has been limited success in developing disease modifying therapies. Most work thus far has been focused on CNS-centric therapies, a logical starting place. But, through the search for biomarkers and risk factors, accumulating evidence shows that lifestyle, diet, and overall health (modifiable risk factors) have a larger impact on disease incidence than initially thought. Understanding the role GI dysfunction likely plays in neurodegenerative disease risk may aid in earlier diagnosis and/or more patient specialized therapeutic interventions for those with increased gut barrier permeability. If this periphery-focused hypothesis is correct, it opens a large number of opportunities to target the microbiome to prevent or reverse dysbiosis as a way to delay, arrest, or prevent onset and progression of neurodegenerative diseases. To make this a feasible therapeutic strategy that can be translated on a wide scale, we first need to identify microbiome signatures that can assist the field to predict risk, stage disease, and which can be used to monitor target engagement in clinical interventions aimed at the gut microbiome to modulate brain health. Here, we address what is currently known about how the gut microbiome can modulate the innate immune system in the context of neurodegenerative disorders and clinical trials using microbiota-modulating therapies including probiotics, fecal microbiota transplant (FMT), and dietary interventions.

## Monocytes, Microglia, and the Microbiome

Immune cells are the body's primary defense against pathogens. Microglia and monocytes are innate immune cells and are the first responders when external triggers or stressors (pathogens, xenobiotics, toxicants, etc.) breach the demilitarized zone surrounding the blood-brain and gut-luminal barriers, respectively [9]. Monocytes can differentiate into a plethora of circulating and tissue resident innate immune cells. Because they are often the initial responders to tissue disruption and an initiator of the inflammatory cascade, they are an important population to examine in the context of age-related neurodegeneration and environmental interactions [10]. Monocytes will most often differentiate into either macrophages or dendritic cells, professional phagocytes and antigen presenting cells, respectively. The tissue resident cells tend to be long-lived while circulating monocytes are typically only active for 2–3 days. Their three main functions are phagocytosis, antigen presentation, and cytokine release [10]. The microbiota modulate the activity of bone marrow-derived circulating monocytes and microglia via a complex network of metabolites, the normal function of which the field is currently aiming to unravel [[11], [12], [13], [14]].

Microglia are the tissue-resident macrophages of the brain parenchyma, differing from peripheral tissue-resident macrophages in their origin and functions. Microglia are derived from the yolk sac during development and are self-renewing throughout life [15]. Microglia initiate inflammatory cascades in response to injury, infection, and other pathology and thus are of special interest in the context of neurodegenerative disease. Characterization of microglial phenotype throughout the human lifespan has revealed these cells are especially susceptible to age- and environmental-related changes ranging from gene expression, morphology, and activity [16]. There is also evidence illustrating that hematopoietic derived macrophages exist in the brain, especially during disease, but they are not yet well described [17]. Trafficking of macrophages to the brain and maturation of the microglia are influenced by exposure to the environment, specifically through the gut microbiota [18,19].

The gut microenvironment is highly complex. This living ecosystem not only consists of viruses, bacteria, and fungi, living in the gastrointestinal tracts, but also their secretomes, metabolites, and waste. Many of these molecules are immunogenic and therefore have the potential to be sensed by the human host and influence host physiology. Direct methods of communication between the gut microbial community and the CNS are via the enteric nervous system (ENS) and vagus nerve. The ENS is composed of sympathetic neurons, glia, and ganglia that innervate the intestines and stomach [20]. Enteroendocrine cells (EECs) have the capacity to synapse directly to the ENS which allows for direct signaling through the vagus nerve [20]. Beyond direct synaptic connections, the digestive tract and the brain are intimately linked by hormonal signaling via the diet and metabolites via energy metabolism. For example, the brain utilizes the most calories of all organs by a substantial margin, thus, brain function is critically linked with energy availability.

**Fig. 1:**
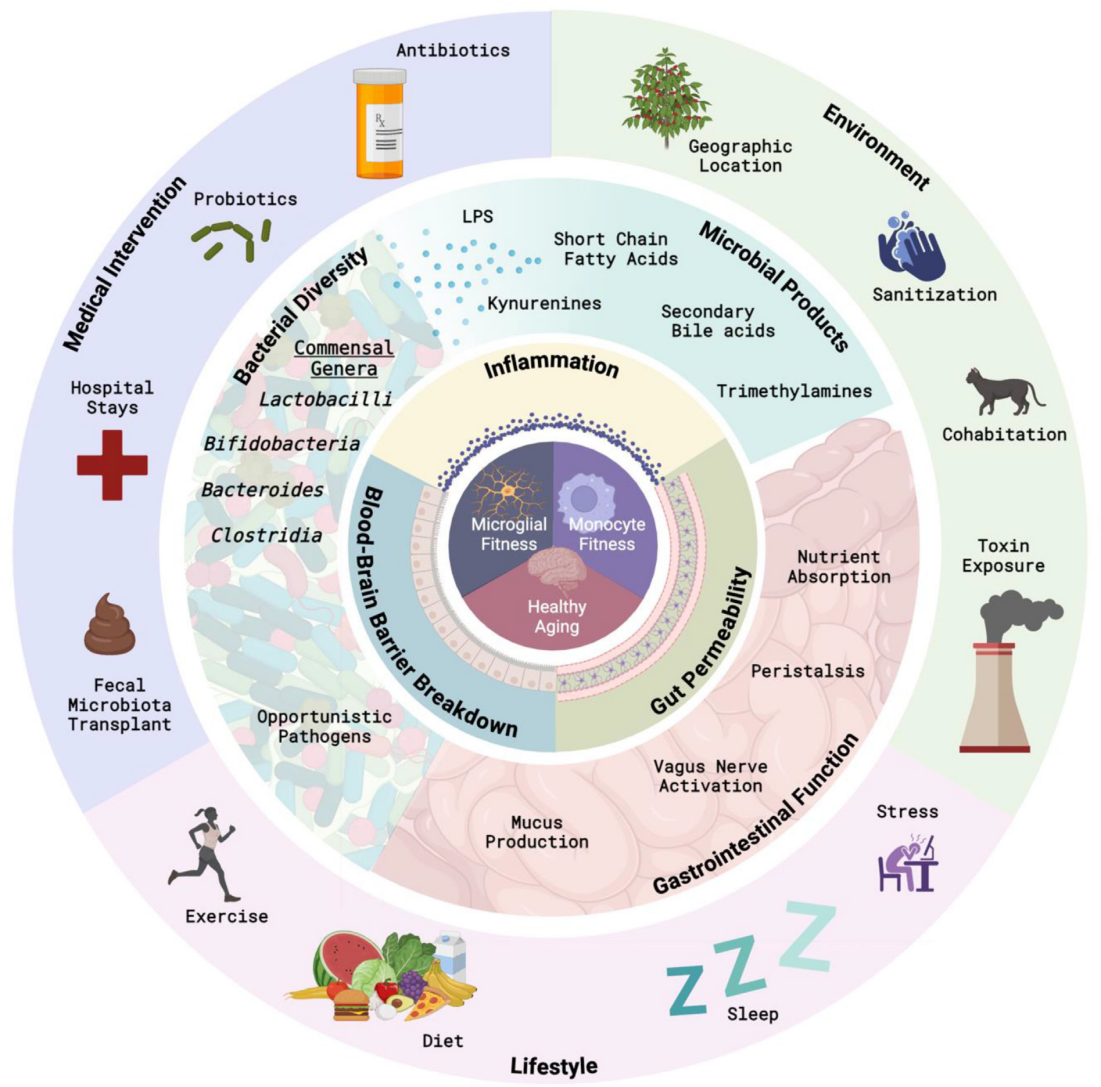
**Brain health is dependent on microglial and peripheral monocyte fitness which are mediated by different degrees of environmental exposure**. Lifestyle, environment and medical intervention all modulate the gut microenvironment. Stable, diverse bacterial communities producing immune modulating microbial products and stable gastrointestinal function further influence the body's barriers. The gut barrier, the blood-brain barrier, and the immune system all operate to protect the host from damage and pathogens, but dysregulation can lead to excess stress that accumulates over time leading to neurodegeneration. Overall, each ring represents a collection of factors that together determine the outcomes in the subsequent smaller rings, emphasizing the complex interactions influencing aging and development of neurodegenerative diseases.

The communication between the brain and the gut immune system is an often-overlooked connection that influences brain health, however this signaling highway is likely the most important when examining gut-based therapies and interventions for neurological disorders. The gut immune system is robust and its regulation is tightly controlled due to the constant interface with exogenous environmental- and bacterial -derived antigens. Here, we will describe current gut microbiota-modifying methods being tested as disease modifying therapies in neurodegenerative disorders (Fig. 1).

## Microbial-Derived Immune Mediators

One of the most studied immune mediating microbial metabolites are the diverse family of short chain fatty acids (SCFAs). Consumption of a well-rounded high fiber diet or supplementation with the prebiotic microbiota-accessible carbohydrates (MACs) increases the production of SCFAs [21]. SCFAs can reduce inflammation within the colon and elsewhere as they are absorbed into circulation from the gut mucosa [22]. SCFAs also have the capacity to cross the BBB and directly interact with microglia [23]. They are ligands for the G protein coupled receptors free fatty acid receptor 2 and 3 (FFAR2/3) which are present on monocytes and macrophages among other cell types [[24], [25], [26], [27]], indicating the role SCFAs can play in monocyte and macrophage regulation. Additionally, monocyte cell lines have the ability to reduce IL-1β and TNF after exposure to SCFAs [28]. In addition to signaling through GPCRs, SCFAs are known to exert systemic effects through the inhibition of histone deacetylase (HDAC) activity. Butyrate has specifically been shown to increase the antimicrobial activity of macrophages through its inhibition of HDAC3 [29]. Recent work has demonstrated reduced levels of butyrate and propionate in the stool of individuals with Parkinson's disease (PD) compared to healthy controls [30]. Furthermore, reduced levels of butyrate are associated with epigenetic changes in leukocytes and neurons in people with PD [31].

Tryptophan is an essential aromatic amino acid whose metabolites (including kynurenines, indols, and serotonin) can also mediate immune function. Species of *Lactobacillus, Peptostreptococcus, Clostridium* and likely many others can metabolize tryptophan into kynurenines [[32], [33], [34]]. They produce the bioactive kynurenic acid, quinolinic acid, and 3-hydroxykynurenine. The majority of the kynurenic acid used by the body is produced in the digestive system with highest concentrations near the colon [35]. Kynurenines can also be produced by monocyte-derived macrophages in response to interferon stimulation [36,37]. Conversely, kynurenine can contribute to macrophage activation through an increase in CCL2-mediated migration by monocytes that can target most tissues, including the brain [38]. Like kynurenine, quinolinic acid and 3-hydroxykynurenin can also cross the blood-brain barrier, where they can act to increase oxidative stress and signaling [39]. These bacterially derived metabolites are bioavailable in the CNS and can drive inflammation. Individuals with neurodegeneration including Alzheimer's disease (AD), multiple sclerosis (MS), Huntington's disease, amyotrophic lateral sclerosis have dysregulated kynurenine [40]. Further, in individuals with PD, kynurenine levels are higher than healthy controls and have been shown to be reduced after treatment [41]. The kynurenine family of metabolites are associated with systemic inflammation and their ability to cross the blood brain barrier makes them a therapeutic target for neurodegeneration.

Trimethylamines are microbial metabolites produced primarily by *Anaerococcus, Clostridium, Escherichia, Proteus, Providencia*, and *Edwardsiella* [42]. They result from the breakdown of choline and carnitine found in dairy products, meats, and fish [43]. They have primarily been studied in the context of cardiovascular disease [44]. In 2017, however, Del Rio et al. found trimethylamine-N-oxide in the cerebrospinal fluid (CSF) so it is believed to be able to cross the BBB. While they found that trimethylamine levels in the CSF did not correlate with dementia or AD [45], others have suggested increased levels may serve as a biomarker of AD [46]. Circulating levels of trimethylamines tend to increase with age in mice. In mouse models of AD, increased levels lead to changes in pathological hallmarks like increased b-secretase and b-secretase-cleaved C-terminal fragment (bCTF) in the hippocampus [47]. In vitro*,* trimethylamine-N-oxide promotes microtubule assembly of mutant tau protein [48], which is a foundational feature of AD, indicating it may contribute to AD pathology [49]. Many of the bacterial groups found to increase in people with MS produce trimethylamines, but no direct relationship has yet been drawn [50]. In a mouse model of PD, trimethylamines lead to increased microglia activation as well as increased circulating TNF and IL-1 [51,52]. Trimethylamines and other gut microbiota derived molecules are correlated with neurodegeneration; however, the underlying biological mechanisms are still not well understood.

New microbiota derived bioactive molecules are regularly being discovered. Still more have been identified, but their mechanisms are not yet described. Some of these include secondary bile acids, equols, and anthocyanins. For an in-depth review of the effects of microbiota-derived mediators on monocytes the authors refer the reader to the following review [53].

## Disease Modifying Therapies

### Diet

One of the most intimate ways we interact with our environment is through the food that we choose to consume. The diet one consumes provides energy sources, nutrients, and minerals that we need to continue to grow and be active. However, the diet also shapes the function, structure, and overall make-up of the gut microbiota. The nutrients ingested from food directly catalyze growth of specific bacteria, which in turn produce bioactive metabolites that can aid in either maintenance or breakdown the gut barrier [54].

Diets high in fat and sugar have been demonstrated to be detrimental to neuronal health in aging and neurodegeneration. The mechanism of cognitive decline due to high-fat diet is currently under debate, but a leading hypothesis is an increase of inflammation as a result of chronic exposure to a Western diet. In rodent models, high-fat diets cause a decrease in the alpha diversity of the gut microbiota resulting in overall reduced abundances of *Firmicutes* and *Bacteroidota* [55,56]. This change may lead to increased gut permeability, which in turn leads to increased systemic inflammation, increased neuroinflammation, and poor cognitive aging [57]. This effects memory, social behavior, as well as motor and sensory dysfunction with age [58,59].

Diets high in fiber have been shown to produce SCFAs. As described earlier, SCFAs help protect from neuroinflammation. The bacterial groups that seem to drive this change include many fermenting bacteria including *Bacteroidetes, Fecalibacterium, Bifidobacterium, and Lactobacillus* with lower abundances of *Firmicutes* and *Proteobacteria* [60,61]*.* High levels of fiber are characteristic of several lifestyle diets including the Mediterranean diet. The Mediterranean diet is characterized as plant-based diet that emphasizes consumption of fruits, vegetables, legumes, and vegetable oils, while limiting sugars and refined carbohydrates. Based on a recent meta-review, an adherence to a Mediterranean diet will reduce the incidence of PD and AD, however, there are some inherent flaws in the conclusions of this work [62]. Unfortunately, based on the available data, we are unable to control for a variety of confounding factors in these longitudinal studies including geographic location, cultural differences, and genetic differences when studying these dietary populations. The studies tend to be small and rely on self-reporting observations. The positive benefits of the Mediterranean diet are traditionally attributed to the anti-inflammatory and antioxidant effects of the polyphenols and omega-3 fatty acids. However, because this diet is also rich in fiber, it is also likely that this diet also promotes inflammatory modulation through maintenance of an anti-inflammatory gut microbiota and reduced gut permeability.

A new, more prescribed form of the Mediterranean diet is called the Mediterranean Dietary Approach to Stop Hypertension Intervention for Neurodegeneration Delay or MIND diet and is hypothesized to help with healthy cognitive aging [63]. Beyond the traditional Mediterranean diet, the MIND diet also includes low-fat dairy, whole grains, nuts, and legumes [64]. While preliminary evidence has shown that this diet can delay cognitive decline, a characterization of its effects on the gut microbiota has yet to be completed.

The ketogenic diet is another popular lifestyle diet that is used to treat some forms of epilepsy [65]. While its benefits in epilepsy are well-established, the benefits of the ketogenic diet in normal aging are less robust. The ketogenic diet is characterized by an extreme reduction in carbohydrate intake and an increase in fats-usually from animal products. This leads to increases in gut resident *Ruminococcaceae* and decreases in *Prevotellaceae, Bacteroidaceae, Acidaminococcaceae, Rikenellaceae,* and *Sutterellaceae* [66]. Studies examining the role of the ketogenic diet have been conflicting and there is little evidence to suggest that it will be useful in prevention of neurodegeneration in the wider public [67].

With all these diets, larger longitudinal studies are still necessary to establish the specific effects of diet on neurodegeneration. Optimal diets and resulting nutrition will and should be unique to everyone, however, a communal emphasis on diverse, fiber-rich diets can be encouraged in order to help promote a stable and healthy microbiome.

### Pre-/pro-biotics

Prebiotics are a substrate leveraged by microorganisms to catalyze a positive health outcome [68]. Fructo-oligosaccharides (FOS), galacto-oligosaccharides (GOS), lactulose, and inulin are the most widely recognized prebiotics [69]. Prebiotics can be consumed through fruits and vegetables, which are then fermented and broken down to SCFAs by the gut microbiota [70,71]. The use of prebiotics has been shown to modulate stress, anxiety, and hippocampal synaptic efficacy in animal models of disease [70]; yet there is minimal literature demonstrating that prebiotics can modify risk or progression of neurodegenerative disease.

Probiotics are living organisms consumed to confer a health benefit on the host [72]. The seven core genera that are most commonly used in probiotics are *Lactobacillus, Bifidobacterium, Saccharomyces, Streptococcus, Enterococcus, Escherichia*, and *Bacillus* [73]. A key role of probiotic bacteria is immunomodulation. Recent data have indicated probiotics may help to restore gastrointestinal function and ameliorate inflammation. For example, macrophage phagocytosis increased following pre-treatment with several *Lactobacillus* species [74,75]. Moreover, a recent meta-analysis revealed that gene expression of IL-1, IL-8 and TNF were downregulated in response to probiotic intake in people with PD [76]. While there is increasing evidence that probiotics have beneficial effects, data from ongoing and future clinical trials will be needed to determine if they can be used as disease modifying therapeutics in the context of neurodegenerative diseases. The current body of clinical trials will be further discussed in this review.

### Fecal microbiota transplant

Fecal microbiota transplant (FMT) is a process by which the microbiota from one donor is transferred into another. There are several methods for conducting the transplantation. Usually, the recipient begins with several rounds of antibiotics to disrupt the native microflora, opening a niche for the donor community. The donor community can be delivered during a colonoscopy, via a nasal duodenal tube, via an enema, or through an oral capsule. FMT is generally regarded as safe and well-tolerated, however, it is important that proper donor screening is done to ensure the absence of pathogens to reduce the risk of infection [77]. There is also concern of the longevity of the donor community, thus, often patients may undergo multiple rounds of FMT. The difficulty establishing permanent microbial population changes also means that treating chronic conditions with FMT is challenging. Overall, despite fears surrounding the transfer of pathogens, this procedure has been found to be safe, even in elderly and compromised patients. This method is most frequently used to treat *Clostridium difficile* infection, however recent observations have indicated the possible benefits of FMT on restoring gut microbial diversity of individuals with co-occurring neurodegeneration, thus driving the use of FMT as a candidate for treatment of neurological disorders [78].

## Gut-Brain-Axis in Immune Mediated Disorders

### Gastrointestinal disorders

Irritable bowel disease (IBD), constipation, and other GI symptoms are well characterized precursors and/or risk factors for several neurodegenerative diseases including PD, AD and related dementias (ADRDs), and MS [[79], [80], [81]]. Although no current therapy directly targets monocyte activity in individuals living with GI dysfunction and resulting gut inflammation, current commonly used anti-inflammatory therapeutics like aminosalicylates, biologics (i.e. anti-TNF or anti-integrin), corticosteroid and antibiotic therapies can modulate monocyte activity [82]. Gut resident macrophages play a critical role in regulating homeostasis or lack thereof in the gut [83]. When the gut is inflamed, blood monocytes will migrate to the gut barrier to aid in the inflammatory response and transition into pathogen defense-associated intestinal macrophages [82]. In fact, individuals with Crohn's disease (CD) (a type of GI dysfunction characterized by remittent inflammation of bowels) generally have fewer blood monocytes with an increase in total intestinal macrophages [84], indicating a migration of monocytes from the blood to the gut during inflammation. In a recent study evaluating people with GI dysfunction, evidence of monocytosis was able to predict disease severity and activity—leading the authors to conclude that monocyte infiltration into the gut during disease may be an indicator of inflammation and overall disease outcome [85]. Additionally, patients with monocytosis had worsened clinical outcomes, resulting in worsened reports of quality of life and drastically increased emergency department visits, surgery, and hospitalization rates. Due to the powerful yet heterogenous role monocytes play in the GI circuit, there is still no clear-cut explanation for the causative role monocytes may play in catalyzing disease—warranting continued research and investigation.

Further, individuals with GI symptoms and those diagnosed with IBD have disruptions in their gut microbiomes, termed dysbiosis [86]. These individuals have decreased SCFA producing gut bacteria and increases in *E. coli* as well as other pro-inflammatory bacteria [87,88].

#### Probiotics

The use of probiotics as a therapeutic strategy for IBD has shown mixed results, however, it is important to note that these differences in outcomes may be linked to the heterogeneity in study design (intervention duration, strain of probiotic, etc.). When people with IBD were treated with *Bacillus clausii* UBBC-07, alterations in the gut microbiome were reported along with modulation of disease symptoms [89]. Specifically, a significant increase in *Lactobacillus*, *Bifidobacterium* and *Faecalibacterium* were observed in the probiotic treated group compared to the placebo group. Moreover, in the group with ulcerative colitis (UC), Simple Clinical Colitis Activity Index (SCCAI) scores were significantly decreased, indicating an improvement in UC symptoms with probiotic treatment. However, there was no significant decrease in Crohn's Disease Activity Index (CDAI) scores in the CD group. Probiotics have been shown to improve the quality of life of individuals with IBD. When people with UC were treated with probiotics (containing nine *Lactobacillus* and five *Bifidobacterium* species) for six weeks, quality of life scores were significantly improved [90].

In another recent study, a multi-strain probiotic (containing *Lactobacillus rhamnosus*, *Lactobacillus plantarum*, *Lactobacillus acidophilus* and *Enterococcus faecium*) was shown to significantly reduce fecal calprotectin levels in individuals with UC but not in those with CD [91]. However, there were no reported differences in-quality of-life scores between the placebo and probiotic groups.

The beneficial effects of probiotic interventions observed in these clinical trials may be, at least in part, attributed to their effects on monocyte and macrophage populations. For example, calprotectin is produced by monocytes under inflammatory conditions [92]. Thus, the reduction in calprotectin levels following probiotic intervention may be due to anti-inflammatory effects on monocytes. Moreover, it is possible that the improvement in symptoms and quality of life scores is a result of anti-inflammatory effects of probiotics on GI-resident monocytes and macrophages.

#### Fecal microbiota transplant

Several FMT studies have demonstrated the potential benefit of this therapeutic approach for treating IBD [[93], [94], [95]]. Particularly, the efficacy of FMT for maintaining remission of IBD has been studied. A recent pilot study in CD patients in remission demonstrated one FMT from healthy donors was sufficient to improve clinical measures including Crohn's Disease Endoscopic Index of Severity (CDEIS) scores and CRP levels, however, the single FMT was not sufficient to alter the microbiome six weeks later [96]. Similarly, a study of individuals with UC found that FMT induced remission in significantly more people than placebo [97]. Moreover, those that received FMT had greater microbial diversity. Despite many positive reports regarding the efficacy of FMT, several studies have found FMT to be ineffective. For example, a recent 2023 study showed that 12 months post-FMT there was no difference in maintenance of remission between the FMT group and placebo group [98]. Importantly, the discrepancies reported in the outcomes of these studies are likely due to the heterogeneity in approach.

In a recent study of people with UC, several bacteria and microbiota metabolites were found to be associated with FMT induced remission [99]. Individuals in remission following FMT had enrichment of *Eubacterium hallii* and *Roseburia inulivorans*. Short chain fatty acid synthesis and secondary bile acids were also increased in people who achieved remission. Moreover, those that did not achieve remission displayed enrichment of *Fusobacterium gonidiaformans*, *Sutterella wadsworthensis*, and *Escherichia* species.

### Parkinson's disease

Parkinson's disease (PD) is characterized by age-related motor deterioration and neurodegeneration. A component of PD etiopathogenesis is peripheral immune cell infiltration into the brain, where chronic proinflammatory immune activity has been shown to be a fundamental catalyst of PD. Monocytes, among other immune related cells, present antigens to catalyze an immune response to pathogens. Specific MHC-II antigen presentation complexes show increased expression on monocytes derived from people with PD compared to neurologically healthy controls [100]—indicating an ability for monocytes to participate in and convey the immunologic abnormalities of PD.

A new wave of literature provides evidence that PD is a multisystem disorder, hinging on crosstalk between the brain and the peripheral immune system. Several recent studies have detailed the modified metabolism of peripheral blood monocytes from individuals with PD, and have identified a distinct correlation between PD duration and mitochondrial energy capacity in peripheral blood mononuclear cells (PBMCs) [101]. The use of PBMC gene expression status has also emerged as a budding site for biomarkers in PD. PBMCs reveal increases in inflammation and adaptive immune cell response associated gene expression [102]. The authors direct the reader to Karaaslan et al., 2021 for a detailed list of all blood immune-cell transcriptomic studies and results in individuals with PD [102].

Alpha-synuclein (α-syn) is one of the main neuropathological hallmarks of PD. α-Syn is expressed in several immune cell types and has been shown to increase in monocytes following an inflammatory stimulus [103]. PBMCs have a correlational increase in oligomeric α-syn deposition and accumulation of additional autophagy related proteins with disease duration [104], which is supported by similar findings of autophagic protein accumulation in PD brains [105]. Additionally, cholinergic neurons can reflexively modulate the acute inflammatory response induced by inappropriate microbe invasion through the vagus nerve [106]. This anti-inflammatory reflex drives the modulation of inflammatory mediators via peripheral immune cells to prevent further invasion of inflammatory triggers into the bloodstream [107]. Outside of cholinergic neurons but still within the complex catecholaminergic signaling system, the expression signature of dopamine transporter (DAT) and tyrosine hydroxylase (TH) is distinct in PD peripheral immune cells, corroborating the association between dopaminergic consequences in the CNS and the peripheral immune system [108]. These findings allude to the role monocytes can play in catalyzing the inflammatory cascade involved in PD pathogenesis. In parallel with the inflammatory changes, individuals with PD often display dysbiosis characterized by increased *Bifidobacterium* and *Hugatella* among others and decreased *Roseburia, Faecalibacterium,* and *Lachnospiraceae* [[109], [110], [111], [112], [113]]*.* Overall, these trends indicate that the microbiota and immune system are changing with PD progression and may be a point for intervention to slow down progression.

Several meta-analyses have identified a correlation between GI dysfunction and PD risk. Constipation is the most common GI symptom associated with PD and can occur 15.6–24 years prior to the onset of motor features [114,115]. One study identified that middle-aged males who had less than one bowel movement a day had a four-fold increased risk for PD development over a 24 year period, as compared to age and sex-matched counterparts with more regular regimens of bowel movements [116]. Additionally, several studies have identified an increase in intestinal wall permeability of people with PD, as compared to age and sex-matched controls [117,118]. However, this increased permeability was in the absence of gross intestinal architecture morphological change or damage – indicating the possibility for low-grade but chronic levels of peripheral inflammation in these individuals. An added complexity of gut-brain interaction in patients with PD is that the most common therapy for PD-induced motor symptoms, levodopa, is metabolized differently by different commensal gut microbes. *Enterococcus faecalis, Eggerthella lenta,* and *Helicobacter pylori* among others can impair the clinical response to levodopa through differential metabolism [119].

Gut-associated proinflammatory triggers in human studies that have been correlationally linked to PD risk include pesticide or toxic pollutant exposure [120] as well as infection [121]. An additional and much more frequent inflammatory stimulus, especially in the United States, is IBD. In parallel to the rise in PD diagnoses, IBD diagnoses have increased 46 ​% from 2006 to 2021—with the primary justification being change in diet makeup and environmental toxicant exposure [122]. The exposure to these toxic pollutants and/or an infection that modulates the gut flora are hypothesized to be the opening catalyst to chronic inflammation and concomitant peripheral immune dysfunction.

#### Diet

A plethora of anecdotal and observational studies have shown promise to reverse symptom progression in people with PD. In fact, a long-term longitudinal observational study of over 150,000 Americans found that plant-based dietary patterns low in saturated fats may protect against PD [123]. Unfortunately, randomized controlled trials are less common. A single randomized controlled trial has been conducted to test a Mediterranean diet in 80 people with PD. After ten weeks, participants had lower scores on the Movement Disorders Society-Unified Parkinson's Disease Rating Scale (MDS-UPDRS) and reduced cognitive impairment [124,125]. Four randomized controlled trials have tested the ketogenic diet in PD patients. While they are all pilot studies with low numbers of participants, none have shown improvement to motor symptoms associated with PD, however, they do report compelling evidence of improved quality of life [[126], [127], [128], [129]]. Finally, individuals with PD that were put on a two-week ovo-lacto vegetarian style diet had significantly decreased UPDRS III scores one year after dietary intervention [130]. When this dietary intervention was combined with bowel cleansing, UPDRS III scores were reduced even further, and levodopa dosage was also decreased one-year post-intervention. In addition to these dietary interventions, there is mounting evidence that Vitamin E, Vitamin C, and omega-3 fatty acid supplements can improve motor function in people with PD [[131], [132], [133]].

#### Probiotics

The use of probiotics for treatment of PD and other neurodegenerative diseases has recently been gaining additional attention. Several studies have been conducted examining the effectiveness of probiotics in relieving GI symptoms associated with PD. For example, a 2016 study by Barichella et al. demonstrated that after 4 weeks of consuming fermented milk containing multiple probiotic strains and prebiotic fiber resulted in an increase in complete bowel movements in people with PD compared to those taking a placebo [134]. In PD patients with constipation, treatment with probiotics containing *Lactobacillus acidophilus* and *Bifidobacterium infantis* twice per day 1 ​h after meals for 3 months resulted in improved GI symptoms including abdominal pain and bloating [135]. However, this probiotic intervention was not as effective as trimebutine in relieving constipation. A 2021 study by Tan and colleagues demonstrated that 4-week intervention of multi-strain probiotic intake increased spontaneous bowel movements by 1.0 ​± ​1.2 per week in individuals with PD compared to a decrease by 0.3 ​± ​1.0 in the placebo group [136]. Whole-gut transit time has also shown to be improved with probiotic intervention in people with PD with constipation [137].

In addition to GI symptoms, probiotics may influence other PD symptoms. A 12-week intervention of daily probiotic intake, containing *Lactobacillus acidophilus, Bifidobacterium bifidum, L. reuteri, and L. fermentum*, resulted in a significant decrease in MDS-UPDRS scores in people with PD [138]. Conversely, PD patients treated with Hexbio® showed no significant difference in MDS-UPDRS part II or MDS-UPDRS part III scores compared to those individuals in the placebo group [137].

In addition to examining the effects of probiotics on clinical symptoms, several groups have aimed to investigate the effects of probiotic intervention on peripheral inflammation. For example, a 2018 study revealed that following 12 weeks of probiotic treatment (8 ​× ​10^9^ ​CFU/day), gene expression of IL-1, IL-8, and TNF were downregulated in PBMCs from individuals with PD compared to those in the placebo group [139], indicating a potential beneficial anti-inflammatory effect of probiotics on peripheral blood immune cells.

As outlined above, many studies have investigated the effects of probiotics on PD symptoms, however, the effects of these interventions on the microbiome of people with PD have not been extensively studied. A recent study examined the effects of 12-week probiotic treatment (containing *Bacillus licheniformis, Lactobacillus acidophilus*, *Bifidobacterium longum*, *Enterococcus faecalis*) on constipation and the gut microbiome of people with PD [140]. The results of this study indicate that probiotic treatment not only can improve GI symptoms, but also, and unsurprisingly, alter the composition of the gut microbiome. 16s RNA sequencing revealed *Christensenella Marseille* P2437 increased significantly following probiotic treatment, while *Eubacterium oxidoreducens*, *Eubacterium hallii,* and *Odoribacter* N54.MGS-14 all decreased. Interestingly, the results of this study indicate a negative correlation between *Odoribacter N54.MGS-14* and spontaneous bowel movements. Similarly, following 12-weeks of *Lacticaseibacillus paracasei* strain Shirota supplementation, constipation-related symptoms and quality of life scores were significantly improved in people with PD [141]. This treatment paradigm also resulted in an increase in *Lacticaseibacillus* in the microbiome of the probiotic treated group. Targeted metabolomics of 111 fecal metabolites showed no significant differences between all groups; however, l-tyrosine, 3,4-dihydroxyhydrocinnamic acid, and glyceric acid were changed after intervention only in the probiotic group.

Many studies have been performed investigating the effects of probiotic interventions on ameliorating GI symptoms in people with PD, but further investigation into the effectiveness of probiotics on modifying disease progression and how this may be conferred through modulation of myeloid cells is needed.

#### Fecal microbiota transplant

FMT is used in the treatment of several GI diseases and has recently been suggested as a therapeutic strategy for neurodegenerative diseases including PD. A case study of six individuals with PD demonstrated that FMT was safe and in five individuals, FMT improved motor symptoms and constipation four weeks post-transfer [142]. Another study of 11 people with PD found that following FMT, *Blautia* and *Prevotella* were increased while *Bacteroidetes* was significantly decreased in the gut microbiome [143]. Additionally, PAC-QOL and Wexner constipation scores significantly decreased following FMT.

There have been three double-blind, placebo controlled, randomized clinical trials examining the efficacy of FMT at reducing progression of motor symptoms in PD. Two of them reported mild improvement in the FMT group compared to the placebo group in the MDS-UPDRS [144,145]. Unfortunately, the largest double-blind, placebo controlled, randomized clinical trial of 47 participants with PD, FMT did not lead to motor improvement within the 6 months of follow-up [146]. Of note, in this study, participants in the placebo group ended with higher levodopa equivalent daily dose (LEDD) than the FMT intervention group, indicating that those given placebo needed more medication to reduce movement symptoms. It is possible that with further follow-up there may be evidence of a slowing of symptom progression in this group.

### Alzheimer's disease and related dementias

ADRDs are a broad range of acquired neuropsychiatric disorders that are commonly associated with age. The etiologies of ADRDs are variable and include traumatic brain injury (TBI), proteinopathies, and metabolic disorders. They are often associated with a gradual loss of neurons and no disease-modifying therapies have yet been effective. Overall, the largest risk factor for developing dementia is aging with other risk factors including genetic predisposition, environmental exposures, education, diet, and exercise [147].

There is divergent literature describing the role monocytes play in Alzheimer's disease (AD) progression. A distinctive phenotype of inflammatory monocyte infiltration from the periphery into the brain has been characterized in mouse models of AD [[148], [149], [150]]. However, studies with post-mortem brains have yielded discordant results. In one study that analyzed post-mortem tissue from multiple vulnerable regions of interest in AD, researchers found no difference in the distribution or frequency of monocytes in any AD tissue sample (as compared to neurologically healthy control), concluding that there is minimal if no role of monocytes in the clearance of AD disease pathology or progression [151]. Alternatively, recent studies have identified monocytes and monocyte-derived peripheral cell invasion into the brain of post-mortem tissue from people clinically diagnosed with AD [152]. A separate study phenotypically and metabolically classified monocytes based off brain region in post-mortem tissue from people with AD [153].

In support of the idea that peripheral monocyte activation can worsen dementia, individuals with greater peripheral monocyte activation are more likely to have cognitive decline after a vascular event [154,155]. Beyond circulating monocytes, increased activation in both microglia and border associated macrophages are also correlated with both vascular dementia and AD resulting in neuronal toxicity and synaptic loss [156].

People with dementia have marked changes in their microbiomes compared to healthy controls. But due to the diversity of the human microbiome, a consequence of diet, location, and other factors, studies using different cohorts often find different specific differences among these individuals. Despite this variability, some trends that have emerged are an increase in *Saccharibacteria, Faecalibacterium, and Helicobacter pylori* and a reduction in *Prevotella* and *Ruminococcus* in people with AD [157]. In contrast, the class *Melainabacteria* is increased in people with Frontotemporal Dementia, the class *Alphaproteobacteria* is increased in dementia with Lewy bodies, while vascular dementia is associated with the genera *Eubacterium, Ruminococcus, Veillonella* and *Slakia* [158]*.* Hopefully, with greater attention and access to patient samples, future studies will be able to find trends and bridge the gap in the current understanding of the gut-brain relationship in dementia.

#### Diet

Like all age-related neurodegenerative diseases, a confounding factor of neuronal death are the metabolic and activity changes associated with age. A large portion of people with dementia also have comorbidities like obesity and/or diabetes. Thus, many diet and lifestyle interventions are beneficial for people suffering from cognitive decline both physiologically and cognitively. Mounting evidence suggest that Mediterranean diet and the ketogenic diet are able to slow the progression of cognitive decline [159]. In fact, a specific diet has been developed by researchers called the Mediterranean-DASH (Dietary Approaches to Stop Hypertension) Intervention for Neurodegenerative Delay (MIND) diet. This diet has also shown to slow the progression of cognitive decline in people with AD and mild cognitive impairment (MCI) [63]. Each of these diets emphasizes whole foods, reducing sugar and saturated fat and generally leads to a more diverse and rich community of microbiota. In contrast, some long-term longitudinal studies have shown little correlation with diet and cognitive impairment [160]. Overall, interventions that have been effective tend to be short term with high compliance rates. Lifestyle interventions tend to be difficult to maintain and monitor and so more studies are necessary.

#### Probiotics

There have been nine randomized, double-blind placebo-controlled trials for probiotics in people with mild cognitive impairment or dementia. Three tested *Bifidobacterium breve*, two tested *Lactobacillus plantarum,* one compared *Bifidobacterium* to *Lactobacillus,* two tested a probiotic cocktails, and the last tested a cocktail probiotic paired with selenium supplement [[161], [162], [163], [164], [165], [166], [167]] All but one using only *Bifidobacterium breve* found improvement in cognitive function compared to placebo or selenium control after at least 12 weeks of probiotic treatment. When comparing *Bifidobacterium* to *Lactobacillus, Bifidobacterium* had a stronger effect. Further, *Bifidobacterium breve* supplementation was found to prevent the brain atrophy seen in the placebo group as measured by MRI after 24 weeks of treatment [168].

One trial used an active control using subtherapeutic doses of a probiotic cocktail compared to the therapeutic doses. This study found differences in cortisol and inflammatory markers after 12 weeks, but no differences in the cognitive outcomes [169].

#### Fecal microbiota transplant

No randomized placebo controlled trials have been conducted using FMT in people with AD [170]. However, two case reports and one controlled study tracking cognitive impairment in older adults have been documented. In one, an individual received FMT from an aged donor for an unrelated event but was receiving regular cognitive impairment assessments using the Mini-Mental State Examination (MMSE). Within two months, the individual's scores moved from the “mild cognitive impairment” range to “normal cognition” and continued to improve four months after [171]. Another 90 year old individual received two FMTs from a young donor and had increased scores on the MMSE and the Montreal Cognitive Assessment (MoCA) one week after the first FMT which continued to improve for the four months of follow up [172].

In the single controlled study to date, 10 people with moderate cognitive impairment and *C. difficile* infection were given FMT while another matched cohort of 10 people were given antibiotics. Cognition was tracked using the MMSE and the Clinical Dementia Rating Scale-Sum of Boxes Score (CDR-SB). Those receiving FMT showed improved cognition by both measures while there were no statistical differences in people given antibiotics and their scores slightly worsened in the MMSE three weeks after treatment [173]. Another single-arm clinical trial on five individuals with AD has established safety and confirms the maintenance of cognition in these patients, supporting proceeding with further clinical trials for FMT in people with AD [174].

### Multiple sclerosis

Multiple sclerosis (MS) is a chronic autoimmune disease causing the degradation of myelin in the CNS which results in a range of neurological symptoms. MS is mostly driven by T cell and macrophage pathology in which peripheral immune cells infiltrate the CNS at lesion sites that are unpredictable in nature [175]. They can occur in any white matter of the brain, as well as larger myelin tracts including the optic nerve and the spinal cord. The exact cause of MS is still unknown, but it is widely believed to be a combination of genetic and environmental factors that trigger an abnormal immune response. Current therapies for MS are most often immunosuppressant drugs which result in non-specific immune dampening. These therapies have numerous side effects ranging from increased susceptibility to infection to kidney damage [175]. Because of these drawbacks, and that current therapies are aimed only at slowing progression rather than curing the disorder, safer and more effective therapies with fewer side effects are necessary. As such, careful and detailed examination of the known association between the microbiome and people with MS is needed.

The gut microbiota is known to change in individuals with autoimmune diseases including MS [176]. People with MS tend to have lower abundances of *Clostridia, Bacteroides*, *Parabacteroides, Prevotella*, *Faecalibacterium,* and *Lactobacillus* to name a few [[177], [178], [179]]. They tend to have higher abundances of several genera including *Pseudomonas, Haemophilus, Lautia, Methanobrevibacter,* and *Akkermansia* [180,181]*.* Because of the heterogeneity of microbiota communities between individuals, trends still need to be validated across multiple populations. People with MS also have higher levels of bacterial-derived indols and phenols in their cerebral spinal fluid [182]. There are many hypotheses as to why the gut microbiota and their derived metabolites are changed in these individuals. Likely, there is a combination of several factors including host genetics, autoimmune responses, and diet. Overall, studies are underway to determine whether probiotic supplements could be used in conjunction with current therapies to improve outcomes.

We know that innate immune cell subsets change over the course of an inflammatory immune disease like MS, as shown in the mouse model experimental autoimmune encephalomyelitis (EAE) [183]. Strikingly, *Lactobacillus* mirrors this and is decreased in EAE [184]. There is also evidence that T cells home to the intestines prior to the CNS during EAE [185] providing a means of more direct exposure to the gut microenvironment [186]. Peripheral monocytes migrate into the CNS during EAE and transcriptionally transition into antigen presenting cells within the brain parenchyma [187]. What drives those changes is still unknown. Together these findings point to the gut microbiome as another angle to search for disease modifying and preventative therapies.

#### Diet

Like most dietary interventions, in the field of MS, most studies only measure outcomes in the short-term, usually six months or less. This makes it difficult to interpret the data for long-term degeneration in disorders like MS where progression will continue for decades. Several years of follow-up are needed to fully characterize how diet effects the progression of disease. Here, we will discuss the double-blind placebo-controlled dietary studies in people diagnosed with MS, all of which were performed in conjunction with standard therapies. The most promising was a four-month intervention using a prescribed anti-inflammatory, antioxidant rich diet in conjunction with a probiotic containing 4.5 ​× ​10^11^ ​CFU of *Lactobacillus. casei, Lactobacillus acidophilus, Lactobacillus plantarum, Lactobacillus bulgaricus, Bifidobacterium breve, Bifidobacterium infantis, Bifidobacterium longum*, and *streptococcus thermophiles*, along with 100 ​mg concentrated fructooligosaccharide. Thirty-five individuals across three types of MS (primary-progressive MS, secondary-progressive MS, and progressive-relapsing MS) were placed in the treatment group and thirty-five placed in the control group. Several quality of life measures were tracked including the modified fatigue impact scale (MFIS), global pain scale (GPS), bladder control scale (BLCS), bowel control scale (BWCS), and sexual satisfaction scale (SSS) at baseline and at the end of the trial [188]. Those receiving the intervention displayed improved quality-of-life scores in all assessments.

For a systematic review of diet trials in MS that do not include probiotics, the authors direct readers to Harirchian et al. [189]. Four trials modifying diet including ketogenic diet, plant-based diets, and low-fat diets have led to improved disability scores on the expanded disability status scale (EDSS) [[190], [191], [192], [193]]. By a similar measure, calorie restriction does not lead to an improvement on the EDSS [194]. Another popular dietary intervention is the addition oil supplements. Fish oil, olive oil, omega-3 fatty acids, and primrose oil have all been shown to not change disability scores [193,[195], [196], [197], [198], [199]]. However, all of the studies mentioned and more have reported increases in quality-of-life and/or anti-inflammatory activity in people with MS [[200], [201], [202], [203]]. Diet modification, along with existing therapeutics may increase quality of life, decrease fatigue, and reduce disability. However, more studies are necessary to fully elucidate the impact of dietary interventions on disease progression and how these interventions may impact innate immunity.

#### Probiotics

Like all trials using probiotics discussed before, those used in people with MS have used a variety of strains, techniques, protocols, and outcome measures, making it difficult to draw conclusions about their overall efficacy. An early promising trial used helminths as a supplement in individuals with MS. Helminths are parasitic worms that activate Type II immunity. Theoretically, type II immunity reduces the Type 17 immune activity which drives most of the autoimmune reaction in MS. An early Phase 1 study treated five newly diagnosed MS patients with helminths for three months [204]. After treatment, these individuals had fewer cortical lesions measured by MRI than baseline which then returned after discontinuation of the treatment. Additionally, the individuals had increased serum IL-10 and IL-4 [204], which may be a result of increased cytokine production by monocytes and macrophages. Unfortunately a follow-up study in 71 people with MS showed no difference between the helminth groups and the control group [205]. Larger powered studies will be needed to conclusively establish the benefit of helminth therapy.

A double-blind placebo-controlled clinical trial with 20 individuals per group tested a probiotic cocktail containing *Lactobacillus acidophilus*, *Lactobacillus casei*, *Bifidobacterium bifidum*, and *Lactobacillus fermentum* or placebo for 12 weeks [206]. The people given probiotics had reduced transcriptional levels of proinflammatory cytokines IL-8 and TNF in PBMCs. They had reduced C-reactive protein (CRP) and increased insulin sensitivity and LDL-cholesterol levels. Participants reported improved mood and better general health [207]. Unfortunately, the authors did not conduct any known neurological measures or follow up, making it difficult to draw conclusions on neurological benefits of the intervention.

Another group used a similar study design and probiotic cocktail containing 2 ​× ​10^9^ ​CFU of *Bifidobacterium infantis*, *Bifidobacterium lactis*, *Lactobacillus reuteri*, *Lactobacillus casei, Lactobacillus plantarum* and *Lactobacillus fermentum* [208]*.* Like the previous study, this study showed self-reported improved mood and general health as well as decreased CRP. This study also used the gold standard MS disability measure, the EDSS and found that four months of treatment significantly improved the disability scores by an average of 0.5 units on the 10-unit scale [208]. Together, these studies suggest that probiotic bacterial cocktails may turn out to be beneficial to improve both quality of life and disability in people with MS.

In a study of the yeast probiotic *Saccharomyces boulardii,* 20 participants were given the probiotic and 20 participants given a placebo for four months. Like those studies before, the probiotic resulted in decreased inflammatory markers and decreased CRP. Even further, this probiotic reduced pain intensity and fatigue severity while also improving self-reported general health [209]. More studies for yeast probiotics are necessary to fully understand how they interact with current therapy options and which patient populations will benefit the most.

Finally, there are several non-randomized or non-placebo-controlled interventional trials that have been conducted with probiotics. While unfortunately these cannot provide strong support for the use of probiotics in people with MS, they can still guide trial design and intervention discovery in the future [210]. Overall, more work is needed to understand the interplay between probiotics and MS and the extent to which probiotic interventions might be beneficial in modifying disease symptoms and progression, however, probiotics do improve quality of life in people with MS.

#### Fecal microbiota transplant

Currently 15 people with multiple sclerosis have been given an FMT. Some have received the FMT for explicit treatment of MS, while others have received the FMT to address GI complaints frequent in people with MS. Of those 15 patients, 3 individuals have reported substantial improvement including no longer needing wheel chair assistance or being completely symptom free [78]. Three reported moderate improvement as measured by disability rating scales or reported long-term halting of disease progression [[211], [212], [213]]. Nine individuals have not had sufficient follow-up to determine long-term outcomes [214]. Overall, FMT seems to be well tolerated in people with MS and substantially beneficial in terms of disability progression and quality of life.

## Future Challenges

In the past two decades it has become apparent that brain function and behavior is influenced by peripheral organs and peripheral inflammatory conditions, including gut inflammation. Specifically, brain-resident and peripheral innate immune cells communicate bidirectionally throughout the lifespan of an organism and this crosstalk becomes dysregulated with unhealthy aging, environmental exposures, lack of physical activity, and poor diet. These lifestyle markers also alter the gut microbiome and likely lead to its dysregulation. The gut microbiome has become an early indication of neuroinflammation as metabolites produced by luminal bacteria influence microglia and monocyte function and phenotypes. Maintaining gut microbiome homeostasis may be key to regulating microglial and monocyte functions that become dysregulated in neurodegenerative diseases.

Moving forward within gut-brain axis research, there needs to be a multifaceted approach with 3 critical and well-integrated phases. First, we must address the need to harmonize collection methods and analysis platforms so that data sets can be compared and cross-correlated widely; formation of microbiome working groups across institutions would be greatly beneficial in this respect. Second, researchers must identify the triggers that cause specific bacteria to go out of balance and when exactly this happens in the course of neurodegenerative disease onset and progression. At present, when bacteria are found to be elevated in a disease, it is difficult to conclude that they are driving the disease, as it may represent an adaptive change in the organism to counter underlying dysfunction. Animal models of prodromal disease will need to be utilized to directly interrogate the earliest features of pathogenesis and the role of gut dysbiosis in that process. We must identify microbiome signatures of risk as well as the specific metabolites produced by bacteria that act on the brain. Ideally, these peripheral gut biomarkers could be routinely checked in a doctor's office to identify individuals at high risk for neurodegenerative diseases so that they could be enrolled in interventional clinical trials targeting the gut microbiota and its metabolites. Finally, we must understand sex and ethnic differences in gut microbiome composition and their effects on the immune system which are likely to influence disease risk and responsiveness to interventions. Most studies to date have focused on relatively homogenous populations which hold limited value when often these data are over extrapolated to vastly heterogenous populations around the world. To this end, well-powered randomized controlled trials in diverse populations are necessary to further interrogate the promise of gut-centric approaches to interrupting neurodegeneration. As we stand at the dawn of this field, preliminary evidence provides motivation to unravel the complex interplay between the gut microbiome, innate immune system, and neurodegeneration, urging us to delve deeper into critical questions that could reshape our understating and treatment of these disorders.

## Contributor Information

ARM spearheaded the writing of the review and generated the figure. ARM, MLB, KBM, and MGT wrote and edited the manuscript. KBM and MGT co-organized the submission and correspondence.

## Funding

Partial funding for this work was derived from awards from NIH/NINDS grant RF1NS128800 (MGT), NIH NIA 1RF1AG057247 (MGT), and the joint efforts of The Michael J. Fox Foundation for Parkinson's Research (MJFF) and the Aligning Science Across Parkinson's (ASAP) initiative. For the purpose of open access, the author has applied a CC-BY public copyright license to the Author Accepted Manuscript (AAM) version arising from this submission.

## Declaration of Competing Interest

The authors declare no conflicting interests.

## References

[1] Rezai-Zadeh K., Gate D., Town T. (2009 Dec). CNS infiltration of peripheral immune cells: D-day for neurodegenerative disease?. J Neuroimmune Pharmacol.

[2] Harms A.S., Yang Y.T., Tansey M.G. (2023). Central and peripheral innate and adaptive immunity in Parkinson's disease. Sci Transl Med.

[3] Chassaing B., Kumar M., Baker M.T., Singh V., Vijay-Kumar M. (2014 Sep 1). Mammalian gut immunity. Biomed J.

[4] Korecka A., Arulampalam V. (2012). The gut microbiome: scourge, sentinel or spectator?. J Oral Microbiol.

[5] Vargas-Rodríguez P., Cuenca-Martagón A., Castillo-González J., Serrano-Martínez I., Luque R.M., Delgado M. (2023 Sep 1). Novel therapeutic opportunities for neurodegenerative diseases with mesenchymal stem cells: the focus on modulating the blood-brain barrier. Int J Mol Sci.

[6] Salminen A. (2023 Mar 1). Activation of aryl hydrocarbon receptor (AhR) in Alzheimer's disease: role of tryptophan metabolites generated by gut host-microbiota. J Mol Med (Berl).

[7] Sampson T.R., Debelius J.W., Thron T., Janssen S., Shastri G.G., Ilhan Z.E. (2016 Dec 1). Gut microbiota regulate motor deficits and neuroinflammation in a model of Parkinson's disease. Cell.

[8] Brown G.C., Camacho M., Williams-Gray C.H. (2023 Jul 1). The endotoxin hypothesis of Parkinson's disease. Mov Disord.

[9] Laursen A.L.S., Olesen M.V., Folke J., Brudek T., Knecht L.H., Sotty F. (2024 Mar). Systemic inflammation activates coagulation and immune cell infiltration pathways in brains with propagating α-synuclein fibril aggregates. Mol Cell Neurosci.

[10] Rigamonti A., Villar J., Segura E. (2023 Dec 1). Monocyte differentiation within tissues: a renewed outlook. Trends Immunol.

[11] Aburto M.R., Cryan J.F. (2024 Apr). Gastrointestinal and brain barriers: unlocking gates of communication across the microbiota-gut-brain axis. Nat Rev Gastroenterol Hepatol.

[12] Celorrio M., Shumilov K., Friess S.H. (2024 Feb 1). Gut microbial regulation of innate and adaptive immunity after traumatic brain injury. Neural Regen Res.

[13] Kim C.S. (2024 Jan 1). Roles of diet-associated gut microbial metabolites on brain health: cell-to-cell interactions between gut bacteria and the central nervous system. Adv Nutr.

[14] Jordan C.K.I., Clarke T.B. (2024 Feb 1). How does the microbiota control systemic innate immunity?. Trends Immunol.

[15] Kwon H.S., Koh S.H. (2020 Dec 1). Neuroinflammation in neurodegenerative disorders: the roles of microglia and astrocytes. Transl Neurodegener.

[16] Bachiller S., Jiménez-Ferrer I., Paulus A., Yang Y., Swanberg M., Deierborg T. (2018 Dec 18). Microglia in neurological diseases: a road map to brain-disease dependent-inflammatory response. Front Cell Neurosci.

[17] Brioschi S., Zhou Y., Colonna M. (2020 Jan 15). Brain parenchymal and extraparenchymal macrophages in development, homeostasis, and disease. J Immunol.

[18] Erny D., De Angelis A.L.H., Jaitin D., Wieghofer P., Staszewski O., David E. (2015 Jun 25). Host microbiota constantly control maturation and function of microglia in the CNS. Nat Neurosci.

[19] Möhle L., Mattei D., Heimesaat M.M., Bereswill S., Fischer A., Alutis M. (2016 May 31). Ly6Chi monocytes provide a link between antibiotic-induced changes in gut microbiota and adult hippocampal neurogenesis. Cell Rep.

[20] Montalbán-Rodríguez A., Abalo R., López-Gómez L. (2024 Jan 1). From the gut to the brain: the role of enteric glial cells and their involvement in the pathogenesis of Parkinson's disease. Int J Mol Sci.

[21] Ayakdaş G., Ağagündüz D. (2023 Sep 1). Microbiota-accessible carbohydrates (MACs) as novel gut microbiome modulators in noncommunicable diseases. Heliyon.

[22] Kim C.H. (2021 May 1). Control of lymphocyte functions by gut microbiota-derived short-chain fatty acids. Cell Mol Immunol.

[23] Hasavci D., Blank T. (2022 Aug 22). Age-dependent effects of gut microbiota metabolites on brain resident macrophages. Front Cell Neurosci.

[24] Trompette A., Gollwitzer E.S., Yadava K., Sichelstiel A.K., Sprenger N., Ngom-Bru C. (2014 Feb). Gut microbiota metabolism of dietary fiber influences allergic airway disease and hematopoiesis. Nat Med.

[25] Shimizu H., Masujima Y., Ushiroda C., Mizushima R., Taira S., Ohue-Kitano R. (2019 Dec 1). Dietary short-chain fatty acid intake improves the hepatic metabolic condition via FFAR3. Sci Rep.

[26] Samuel B.S., Shaito A., Motoike T., Rey F.E., Backhed F., Manchester J.K. (2008 Oct 28). Effects of the gut microbiota on host adiposity are modulated by the short-chain fatty-acid binding G protein-coupled receptor, Gpr41. Proc Natl Acad Sci U S A.

[27] Schlatterer K., Peschel A., Kretschmer D. (2021 Dec 2). Short-chain fatty acid and FFAR2 activation - a new option for treating infections?. Front Cell Infect Microbiol.

[28] Wenzel T.J., Gates E.J., Ranger A.L., Klegeris A. (2020 Jun 1). Short-chain fatty acids (SCFAs) alone or in combination regulate select immune functions of microglia-like cells. Mol Cell Neurosci.

[29] Schulthess J., Pandey S., Capitani M., Rue-Albrecht K.C., Arnold I., Franchini F. (2019 Feb 19). The short chain fatty acid butyrate imprints an antimicrobial program in macrophages. Immunity.

[30] Aho V.T.E., Houser M.C., Pereira P.A.B., Chang J., Rudi K., Paulin L. (2021 Dec 1). Relationships of gut microbiota, short-chain fatty acids, inflammation, and the gut barrier in Parkinson's disease. Mol Neurodegener.

[31] Xie A., Ensink E., Li P., Gordevičius J., Marshall L.L., George S. (2022 Aug 1). Bacterial butyrate in Parkinson's disease is linked to epigenetic changes and depressive symptoms. Mov Disord.

[32] Williams B.B., Van Benschoten A.H., Cimermancic P., Donia M.S., Zimmermann M., Taketani M. (2014 Oct 8). Discovery and characterization of gut microbiota decarboxylases that can produce the neurotransmitter tryptamine. Cell Host Microbe.

[33] Lamas B., Richard M.L., Leducq V., Pham H.P., Michel M.L., Da Costa G. (2016 Jun 1). CARD9 impacts colitis by altering gut microbiota metabolism of tryptophan into aryl hydrocarbon receptor ligands. Nat Med.

[34] Wlodarska M., Luo C., Kolde R., d'Hennezel E., Annand J.W., Heim C.E. (2017 Jul 12). Indoleacrylic acid produced by commensal Peptostreptococcus species suppresses inflammation. Cell Host Microbe.

[35] Turski M.P., Turska M., Zgrajka W., Kuc D., Turski W.A. (2009 Jan 30). Presence of kynurenic acid in food and honeybee products. Amino Acids.

[36] Hu B., Hissong B.D., Carlin J.M. (1995 Jul 31). Interleukin-1 enhances indoleamine 2,3-dioxygenase activity by increasing specific mRNA expression in human mononuclear phagocytes. J Interferon Cytokine Res.

[37] Heyes M.P., Chen C.Y., Major E.O., Saito K. (1997 Sep 1). Different kynurenine pathway enzymes limit quinolinic acid formation by various human cell types. Biochem J.

[38] Zang X., Zheng X., Hou Y., Hu M., Wang H., Bao X. (2018 Apr 5). Regulation of proinflammatory monocyte activation by the kynurenine–AhR axis underlies immunometabolic control of depressive behavior in mice. Faseb J.

[39] Fukui S., Schwarcz R., Rapoport S.I., Takada Y., Smith Q.R. (1991). Blood–brain barrier transport of kynurenines: implications for brain synthesis and metabolism. J Neurochem.

[40] Behl T., Kaur I., Sehgal A., Singh S., Bhatia S., Al-Harrasi A. (2021 Jul 1). The footprint of kynurenine pathway in neurodegeneration: janus-faced role in Parkinson's disorder and therapeutic implications. Int J Mol Sci.

[41] Pathak S., Nadar R., Kim S., Liu K., Govindarajulu M., Cook P. (2024 Jan 10). The influence of kynurenine metabolites on neurodegenerative pathologies. Int J Mol Sci.

[42] Romano K.A., Vivas E.I., Amador-Noguez D., Rey F.E. (2015 Mar 17). Intestinal microbiota composition modulates choline bioavailability from diet and accumulation of the proatherogenic metabolite trimethylamine-N-oxide. mBio.

[43] Bennett B.J., Vallim T.Q.D.A., Wang Z., Shih D.M., Meng Y., Gregory J. (2013 Jan 8). Trimethylamine-N-Oxide, a metabolite associated with atherosclerosis, exhibits complex genetic and dietary regulation. Cell Metabol.

[44] Koeth R.A., Wang Z., Levison B.S., Buffa J.A., Org E., Sheehy B.T. (2013 May). Intestinal microbiota metabolism of l-carnitine, a nutrient in red meat, promotes atherosclerosis. Nat Med.

[45] Del Rio D., Zimetti F., Caffarra P., Tassotti M., Bernini F., Brighenti F. (2017 Sep 22). The gut microbial metabolite trimethylamine-N-oxide is present in human cerebrospinal fluid. Nutrients.

[46] Xu R., Wang Q.Q. (2016 Aug 26). Towards understanding brain-gut-microbiome connections in Alzheimer's disease. BMC Syst Biol.

[47] Gao Q., Wang Y., Wang X., Fu S., Zhang X., Wang R.T. (2019 Oct 10). Decreased levels of circulating trimethylamine N-oxide alleviate cognitive and pathological deterioration in transgenic mice: a potential therapeutic approach for Alzheimer's disease. Aging (Albany NY).

[48] Tseng H.C., Graves D.J. (1998 Sep 29). Natural methylamine osmolytes, trimethylamine N-oxide and betaine, increase tau-induced polymerization of microtubules. Biochem Biophys Res Commun.

[49] Drewes G., Ebneth A., Preuss U., Mandelkow E.M., Mandelkow E. (1997 Apr 18). MARK, a novel family of protein kinases that phosphorylate microtubule- associated proteins and trigger microtubule disruption. Cell.

[50] Praveenraj S.S., Sonali S., Anand N., Tousif H.A., Vichitra C., Kalyan M. (2022 Aug 20). The role of a gut microbial-derived metabolite, trimethylamine N-oxide (TMAO), in neurological disorders. Mol Neurobiol.

[51] Qiao C.M., Quan W., Zhou Y., Niu G.Y., Hong H., Wu J. (2023 Sep 1). Orally induced high serum level of trimethylamine N-oxide worsened glial reaction and neuroinflammation on MPTP-induced acute Parkinson's disease model mice. Mol Neurobiol.

[52] Quan W., Qiao C.M., Niu G.Y., Wu J., Zhao L.P., Cui C. (2023 May 1). Trimethylamine N-oxide exacerbates neuroinflammation and motor dysfunction in an acute MPTP mice model of Parkinson's disease. Brain Sci.

[53] Kolypetri P., Weiner H.L. (2023 Oct 1). Monocyte regulation by gut microbial signals. Trends Microbiol.

[54] Zhang P. (2022 Sep 1). Influence of foods and nutrition on the gut microbiome and implications for intestinal health. Int J Mol Sci.

[55] Wang N., Li C., Zhang Z. (2024 Dec 1). Arctigenin ameliorates high-fat diet-induced metabolic disorders by reshaping gut microbiota and modulating GPR/HDAC3 and TLR4/NF-κB pathways. Phytomedicine.

[56] Roy M., Dumay A., Adiba S., Rozes S., Kobayashi S., Paradis V. (2024 Dec 31). Entamoeba muris mitigates metabolic consequences of high-fat diet in mice. Gut Microb.

[57] Di Vincenzo F., Del Gaudio A., Petito V., Lopetuso L.R., Scaldaferri F. (2024 Mar 1). Gut microbiota, intestinal permeability, and systemic inflammation: a narrative review. Intern Emerg Med.

[58] Hong S., Nagayach A., Lu Y., Peng H., Duong Q.V.A., Pham N.B. (2021 Dec 1). A high fat, sugar, and salt Western diet induces motor-muscular and sensory dysfunctions and neurodegeneration in mice during aging: ameliorative action of metformin. CNS Neurosci Ther.

[59] Bao J., Liang Z., Gong X., Yu J., Xiao Y., Liu W. (2022). High fat diet mediates amyloid-β cleaving enzyme 1 phosphorylation and SUMOylation, enhancing cognitive impairment in APP/PS1 mice. J Alzheimers Dis.

[60] Lykkebo C.A., Nguyen K.H., Niklas A.A., Laursen M.F., Bahl M.I., Licht T.R. (2024 Oct). Diet rich in soluble dietary fibres increases excretion of perfluorooctane sulfonic acid (PFOS) in male Sprague-Dawley rats. Food Chem Toxicol.

[61] Portincasa P., Bonfrate L., Vacca M., De Angelis M., Farella I., Lanza E. (2022 Feb 1). Gut microbiota and short chain fatty acids: implications in glucose homeostasis. Int J Mol Sci.

[62] Solch R.J., Aigbogun J.O., Voyiadjis A.G., Talkington G.M., Darensbourg R.M., O'Connell S. (2022 Mar 15). Mediterranean diet adherence, gut microbiota, and Alzheimer's or Parkinson's disease risk: a systematic review. J Neurol Sci.

[63] Morris M.C., Tangney C.C., Wang Y., Sacks F.M., Bennett D.A., Aggarwal N.T. (2015 Sep 1). MIND diet associated with reduced incidence of Alzheimer's disease. Alzheimer's Dementia.

[64] Seago E.R., Davy B.M., Davy K.P., Katz B. (2024 Sep 26). Neuroprotective dietary patterns and longitudinal changes in cognitive function in older adults. J Acad Nutr Diet.

[65] Dyńka D., Kowalcze K., Paziewska A. (2022 Dec 1). The role of ketogenic diet in the treatment of neurological diseases. Nutrients.

[66] Zharikova A.A., Andrianova N.V., Silachev D.N., Nebogatikov V.O., Pevzner I.B., Makievskaya C.I. (2024 Jan). Analysis of the brain transcriptome, microbiome and metabolome in ketogenic diet and experimental stroke. Brain Behav Immun.

[67] Kaviyarasan S., Chung Sia E.L., Retinasamy T., Arulsamy A., Shaikh M.F. (2022 Oct 14). Regulation of gut microbiome by ketogenic diet in neurodegenerative diseases: a molecular crosstalk. Front Aging Neurosci.

[68] Gibson G.R., Hutkins R., Sanders M.E., Prescott S.L., Reimer R.A., Salminen S.J. (2017 Aug 1). Expert consensus document: the International Scientific Association for Probiotics and Prebiotics (ISAPP) consensus statement on the definition and scope of prebiotics. Nat Rev Gastroenterol Hepatol.

[69] Saxami G., Kerezoudi E., Eliopoulos C., Arapoglou D., Kyriacou A. (2023 Oct 8). The gut-organ Axis within the human body: gut dysbiosis and the role of prebiotics. Life.

[70] Cryan J.F., O’riordan K.J., Cowan C.S.M., Sandhu K.V., Bastiaanssen T.F.S., Boehme M. (2019). The microbiota-gut-brain Axis. Physiol Rev.

[71] Peterson C.T. (2020). Dysfunction of the microbiota-gut-brain Axis in neurodegenerative disease: the promise of therapeutic modulation with prebiotics, medicinal herbs, probiotics, and synbiotics. J evidence-based Integr Med.

[72] Hill C., Guarner F., Reid G., Gibson G.R., Merenstein D.J., Pot B. (2014). Expert consensus document. The International Scientific Association for Probiotics and Prebiotics consensus statement on the scope and appropriate use of the term probiotic. Nat Rev Gastroenterol Hepatol.

[73] Probiotics (2023). https://ods.od.nih.gov/factsheets/Probiotics-HealthProfessional/.

[74] Jaffar N., Okinaga T., Nishihara T., Maeda T. (2018 Jul 1). Enhanced phagocytosis of Aggregatibacter actinomycetemcomitans cells by macrophages activated by a probiotic Lactobacillus strain. J Dairy Sci.

[75] Rocha-Ramírez L.M., Pérez-Solano R.A., Castañón-Alonso S.L., Moreno Guerrero S.S., Ramírez Pacheco A., García Garibay M. (2017). Probiotic Lactobacillus strains stimulate the inflammatory response and activate human macrophages. J Immunol Res.

[76] Xiang S., Ji J.L., Li S., Cao X.P., Xu W., Tan L. (2022 Feb 3). Efficacy and safety of probiotics for the treatment of Alzheimer's disease, mild cognitive impairment, and Parkinson's disease: a systematic review and meta-analysis. Front Aging Neurosci.

[77] Antushevich H. (2020 Apr 1). Fecal microbiota transplantation in disease therapy. Clin Chim Acta.

[78] Borody T., Leis S., Campbell J., Torrex M., Nowak A. (2011). Fecal microbiota transplantation (FMT) in multiple sclerosis (MS). Am J Gastroenterol.

[79] Fu P., Gao M., Yung K.K.L. (2020 Feb 5). Association of intestinal disorders with Parkinson's disease and Alzheimer's disease: a systematic review and meta-analysis. ACS Chem Neurosci.

[80] Yusuf F.L.A., Zhu F., Evans C., Fisk J.D., Zhao Y., Marrie R.A. (2024 Jan 1). Gastrointestinal conditions in the multiple sclerosis prodrome. Ann Clin Transl Neurol.

[81] Pellegrini C., Daniele S., Antonioli L., Benvenuti L., D’antongiovanni V., Piccarducci R. (2020 May 2). Prodromal intestinal events in Alzheimer's disease (AD): colonic dysmotility and inflammation are associated with enteric AD-related protein deposition. Int J Mol Sci.

[82] Gren S.T., Grip O. (2016 May 31). Role of monocytes and intestinal macrophages in Crohn's disease and ulcerative colitis. Inflamm Bowel Dis.

[83] Grainger J.R., Konkel J.E., Zangerle-Murray T., Shaw T.N. (2017 Apr 1). Macrophages in gastrointestinal homeostasis and inflammation. Pflügers Archiv.

[84] Thiesen S., Janciauskiene S., Uronen-Hansson H., Agace W., Högerkorp C.M., Spee P. (2014 Nov 8). CD14(hi)HLA-DR(dim) macrophages, with a resemblance to classical blood monocytes, dominate inflamed mucosa in Crohn's disease. J Leukoc Biol.

[85] Anderson A., Cherfane C., Click B., Ramos-Rivers C., Koutroubakis I.E., Hashash J.G. (2022 Jan 1). Monocytosis is a biomarker of severity in inflammatory bowel disease: analysis of a 6-year prospective natural history registry. Inflamm Bowel Dis.

[86] Serrano Fernandez V., Seldas Palomino M., Laredo-Aguilera J.A., Pozuelo-Carrascosa D.P., Carmona-Torres J.M. (2023 Jul 1). High-fiber diet and Crohn's disease: systematic review and meta-analysis. Nutrients.

[87] Nadalian B., Yadegar A., Houri H., Olfatifar M., Shahrokh S., Asadzadeh Aghdaei H. (2021 Apr 1). Prevalence of the pathobiont adherent-invasive Escherichia coli and inflammatory bowel disease: a systematic review and meta-analysis. J Gastroenterol Hepatol.

[88] Zhuang X., Li T., Li M., Huang S., Qiu Y., Feng R. (2019 Oct 18). Systematic review and meta-analysis: short-chain fatty acid characterization in patients with inflammatory bowel disease. Inflamm Bowel Dis.

[89] Bamola V.D., Dubey D., Samanta P., Kedia S., Ahuja V., Madempudi R.S. (2022 Dec 1). Role of a probiotic strain in the modulation of gut microbiota and cytokines in inflammatory bowel disease. Anaerobe.

[90] Rayyan Y.M., Agraib L.M., Alkhatib B., Yamani M.I., Abu-Sneineh A.T., Tayyem R.F. (2023 Oct 1). Does probiotic supplementation improve quality of life in mild-to-moderately active ulcerative colitis patients in Jordan? A secondary outcome of the randomized, double-blind, placebo-controlled study. Eur J Nutr.

[91] Bjarnason I., Sission G., Hayee B.H. (2019 Jun 1). A randomised, double-blind, placebo-controlled trial of a multi-strain probiotic in patients with asymptomatic ulcerative colitis and Crohn's disease. Inflammopharmacology.

[92] Jukic A., Bakiri L., Wagner E.F., Tilg H., Adolph T.E. (2021). Calprotectin: from biomarker to biological function. Gut.

[93] Chen M., Liu X.L., Zhang Y.J., Nie Y.Z., Wu K.C., Shi Y.Q. (2020 Nov 1). Efficacy and safety of fecal microbiota transplantation by washed preparation in patients with moderate to severely active ulcerative colitis. J Dig Dis.

[94] Crothers J.W., Chu N.D., Nguyen L.T.T., Phillips M., Collins C., Fortner K. (2021 Dec 1). Daily, oral FMT for long-term maintenance therapy in ulcerative colitis: results of a single-center, prospective, randomized pilot study. BMC Gastroenterol.

[95] Chen Q., Fan Y., Zhang B., Yan C., Zhang Q., Ke Y. (2023 Jun 15). Capsulized fecal microbiota transplantation induces remission in patients with ulcerative colitis by gut microbial colonization and metabolite regulation. Microbiol Spectr.

[96] Sokol H., Landman C., Seksik P., Berard L., Montil M., Nion-Larmurier I. (2020 Feb 3). Fecal microbiota transplantation to maintain remission in Crohn's disease: a pilot randomized controlled study. Microbiome.

[97] Moayyedi P., Surette M.G., Kim P.T., Libertucci J., Wolfe M., Onischi C. (2015 Jul 1). Fecal microbiota transplantation induces remission in patients with active ulcerative colitis in a randomized controlled trial. Gastroenterology.

[98] Lahtinen P., Jalanka J., Mattila E., Tillonen J., Bergman P., Satokari R. (2023). Fecal microbiota transplantation for the maintenance of remission in patients with ulcerative colitis: a randomized controlled trial. World J Gastroenterol.

[99] Paramsothy S., Nielsen S., Kamm M.A., Deshpande N.P., Faith J.J., Clemente J.C. (2019 Apr 1). Specific bacteria and metabolites associated with response to fecal microbiota transplantation in patients with ulcerative colitis. Gastroenterology.

[100] Fiszer U., Mix E., Fredrikson S., Kostulas V., Link H. (1994). Parkinson's disease and immunological abnormalities: increase of HLA-DR expression on monocytes in cerebrospinal fluid and of CD45RO+ T cells in peripheral blood. Acta Neurol Scand.

[101] Schirinzi T., Salvatori I., Zenuni H., Grillo P., Valle C., Martella G. (2022 Sep 1). Pattern of mitochondrial respiration in peripheral blood cells of patients with Parkinson's disease. Int J Mol Sci.

[102] Karaaslan Z., Kahraman Ö.T., Şanlı E., Ergen H.A., Ulusoy C., Bilgiç B. (2021 Dec 1). Inflammation and regulatory T cell genes are differentially expressed in peripheral blood mononuclear cells of Parkinson's disease patients. Sci Rep.

[103] Tanji K., Mori F., Imaizumi T., Yoshida H., Matsumiya T., Tamo W. (2002). Upregulation of alpha-synuclein by lipopolysaccharide and interleukin-1 in human macrophages. Pathol Int.

[104] Miki Y., Shimoyama S., Kon T., Ueno T., Hayakari R., Tanji K. (2018 Mar 1). Alteration of autophagy-related proteins in peripheral blood mononuclear cells of patients with Parkinson's disease. Neurobiol Aging.

[105] Zhang Y., James M., Middleton F.A., Davis R.L. (2005 Aug 5). Transcriptional analysis of multiple brain regions in Parkinson's disease supports the involvement of specific protein processing, energy metabolism, and signaling pathways, and suggests novel disease mechanisms. Am J Med Genet B Neuropsychiatr Genet.

[106] Tracey K.J. (2002 Dec 26). The inflammatory reflex. Nature.

[107] Borovikova L.V., Ivanova S., Zhang M., Yang H., Botchkina G.I., Watkins L.R. (2000 May 25). Vagus nerve stimulation attenuates the systemic inflammatory response to endotoxin. Nature.

[108] Gopinath A., Mackie P., Hashimi B., Buchanan A.M., Smith A.R., Bouchard R. (2022 Dec 1). DAT and TH expression marks human Parkinson's disease in peripheral immune cells. NPJ Park Dis.

[109] Wallen Z.D., Demirkan A., Twa G., Cohen G., Dean M.N., Standaert D.G. (2022 Dec 1). Metagenomics of Parkinson's disease implicates the gut microbiome in multiple disease mechanisms. Nat Commun.

[110] Wallen Z.D., Appah M., Dean M.N., Sesler C.L., Factor S.A., Molho E. (2020 Jun 12). Characterizing dysbiosis of gut microbiome in PD: evidence for overabundance of opportunistic pathogens. npj Park Dis.

[111] Toh T.S., Chong C.W., Lim S.Y., Bowman J., Cirstea M., Lin C.H. (2022 Jan 1). Gut microbiome in Parkinson's disease: new insights from meta-analysis. Parkinsonism Relat Disorders.

[112] Romano S., Savva G.M., Bedarf J.R., Charles I.G., Hildebrand F., Narbad A. (2021 Mar 10). Meta-analysis of the Parkinson's disease gut microbiome suggests alterations linked to intestinal inflammation. npj Park Dis.

[113] Nishiwaki H., Ito M., Ishida T., Hamaguchi T., Maeda T., Kashihara K. (2020 Sep 1). Meta-analysis of gut dysbiosis in Parkinson's disease. Mov Disord.

[114] Ueki A., Otsuka M. (2004 Oct). Life style risks of Parkinson's disease: association between decreased water intake and constipation. J Neurol.

[115] Savica R., Carlin J.M., Grossardt B.R., Bower J.H., Ahlskog J.E., Maraganore D.M. (2009). Medical records documentation of constipation preceding Parkinson disease: a case-control study. Neurology.

[116] Abbott R.D., Petrovitch H., White L.R., Masaki K.H., Tanner C.M., Curb J.D. (2001 Aug 14). Frequency of bowel movements and the future risk of Parkinson's disease. Neurology.

[117] Salat-Foix D., Tran K., Ranawaya R., Meddings J., Suchowersky O. (2012 Mar 1). Increased intestinal permeability and Parkinson disease patients: chicken or egg?. Can J Neurol Sci.

[118] Forsyth C.B., Shannon K.M., Kordower J.H., Voigt R.M., Shaikh M., Jaglin J.A. (2011 Dec 1). Increased intestinal permeability correlates with sigmoid mucosa alpha-synuclein staining and endotoxin exposure markers in early Parkinson's disease. PLoS One.

[119] He X., Lai Y., Mo C., Zhang Y., Ai P., Xu S. (2024 Jul 1). Association between fecal bile acids and levodopa response in patients with Parkinson's disease. Microorganisms.

[120] Shrestha S., Parks C.G., Umbach D.M., Richards-Barber M., Hofmann J.N., Chen H. (2020 Dec 1). Pesticide use and incident Parkinson's disease in a cohort of farmers and their spouses. Environ Res.

[121] Bu X Le, Wang X., Xiang Y., Shen L.L., Wang Q.H., Liu Y.H. (2015 Aug 1). The association between infectious burden and Parkinson's disease: a case-control study. Parkinsonism Relat Disorders.

[122] Caviglia G.P., Garrone A., Bertolino C., Vanni R., Bretto E., Poshnjari A. (2023 Jan 1). Epidemiology of inflammatory bowel diseases: a population study in a healthcare district of north-west Italy. J Clin Med.

[123] Gao X., Chen H., Fung T.T., Logroscino G., Schwarzschild M.A., Hu F.B. (2007 Nov 1). Prospective study of dietary pattern and risk of Parkinson disease. Am J Clin Nutr.

[124] Paknahad Z., Sheklabadi E., Moravejolahkami A.R., Chitsaz A., Hassanzadeh A. (2022). The effects of Mediterranean diet on severity of disease and serum Total Antioxidant Capacity (TAC) in patients with Parkinson's disease: a single center, randomized controlled trial. Nutr Neurosci.

[125] Paknahad Z., Sheklabadi E., Derakhshan Y., Bagherniya M., Chitsaz A. (2020 May 1). The effect of the Mediterranean diet on cognitive function in patients with Parkinson's disease: a randomized clinical controlled trial. Compl Ther Med.

[126] Krikorian R., Shidler M.D., Summer S.S., Sullivan P.G., Duker A.P., Isaacson R.S. (2019 Jan 1). Nutritional ketosis for mild cognitive impairment in Parkinson's disease: a controlled pilot trial. Clin Park Relat Disord.

[127] Phillips M.C.L., Murtagh D.K.J., Gilbertson L.J., Asztely F.J.S., Lynch C.D.P. (2018 Aug 1). Low-fat versus ketogenic diet in Parkinson's disease: a pilot randomized controlled trial. Mov Disord.

[128] Tidman M.M., White D., White T. (2022 Apr 1). Effects of an low carbohydrate/healthy fat/ketogenic diet on biomarkers of health and symptoms, anxiety and depression in Parkinson's disease: a pilot study. Neurodegener Dis Manag.

[129] Koyuncu H., Fidan V., Toktas H., Binay O., Celik H. (2021 Dec 1). Effect of ketogenic diet versus regular diet on voice quality of patients with Parkinson's disease. Acta Neurol Belg.

[130] Hegelmaier T., Lebbing M., Duscha A., Tomaske L., Tönges L., Holm J.B. (2020 Feb 6). Interventional influence of the intestinal microbiome through dietary intervention and bowel cleansing might improve motor symptoms in Parkinson's disease. Cells.

[131] Nagayama H., Hamamoto M., Ueda M., Nito C., Yamaguchi H., Katayama Y. (2004 Dec). The effect of ascorbic acid on the pharmacokinetics of levodopa in elderly patients with Parkinson disease. Clin Neuropharmacol.

[132] Pantzaris M., Loukaides G., Paraskevis D., Kostaki E.G., Patrikios I. (2021 Nov). Neuroaspis PLP10^TM^, a nutritional formula rich in omega-3 and omega-6 fatty acids with antioxidant vitamins including gamma-tocopherol in early Parkinson's disease: a randomized, double-blind, placebo-controlled trial. Clin Neurol Neurosurg.

[133] Taghizadeh M., Tamtaji O.R., Dadgostar E., Daneshvar Kakhaki R., Bahmani F., Abolhassani J. (2017 Sep 1). The effects of omega-3 fatty acids and vitamin E co-supplementation on clinical and metabolic status in patients with Parkinson's disease: a randomized, double-blind, placebo-controlled trial. Neurochem Int.

[134] Barichella M., Pacchetti C., Bolliri C., Cassani E., Iorio L., Pusani C. (2016 Sep 20). Probiotics and prebiotic fiber for constipation associated with Parkinson disease: an RCT. Neurology.

[135] Georgescu D., Ancusa O.E., Georgescu L.A., Ionita I., Reisz D. (2016 Nov 11). Nonmotor gastrointestinal disorders in older patients with Parkinson's disease: is there hope?. Clin Interv Aging.

[136] Tan A.H., Lim S.Y., Chong K.K., Manap M.A.A.A., Hor J.W., Lim J.L. (2021 Feb 2). Probiotics for constipation in Parkinson disease: a randomized placebo-controlled study. Neurology.

[137] Ibrahim A., Raja Ali R.A., Abdul Manaf M.R., Ahmad N., Tajurruddin F.W., Qin W.Z. (2020 Dec 1). Multi-strain probiotics (Hexbio) containing MCP BCMC strains improved constipation and gut motility in Parkinson's disease: a randomised controlled trial. PLoS One.

[138] Tamtaji O.R., Taghizadeh M., Daneshvar Kakhaki R., Kouchaki E., Bahmani F., Borzabadi S. (2019 Jun 1). Clinical and metabolic response to probiotic administration in people with Parkinson's disease: a randomized, double-blind, placebo-controlled trial. Clin Nutr.

[139] Borzabadi S., Oryan S., Eidi A., Aghadavod E., Reza, Kakhaki D. (2018). The effects of probiotic supplementation on gene expression related to inflammation, insulin and lipid in patients with Parkinson's disease: a randomized, double-blind, placebo-controlled trial. Arch Iran Med.

[140] Du Y., Li Y., Xu X., Li R., Zhang M., Cui Y. (2022 Oct 1). Probiotics for constipation and gut microbiota in Parkinson's disease. Parkinsonism Relat Disorders.

[141] Yang X., He X., Xu S., Zhang Y., Mo C., Lai Y. (2023 Jul 12). Effect of Lacticaseibacillus paracasei strain Shirota supplementation on clinical responses and gut microbiome in Parkinson's disease. Food Funct.

[142] Segal A., Zlotnik Y., Moyal-Atias K., Abuhasira R., Ifergane G. (2021 Aug 1). Fecal microbiota transplant as a potential treatment for Parkinson's disease - a case series. Clin Neurol Neurosurg.

[143] yi Kuai X., han Yao X., juan Xu L., Zhou Y qing, Zhang L ping, Liu Y. (2021 Dec 1). Evaluation of fecal microbiota transplantation in Parkinson's disease patients with constipation. Microb Cell Factories.

[144] Cheng Y., Tan G., Zhu Q., Wang C., Ruan G., Ying S. (2023). Efficacy of fecal microbiota transplantation in patients with Parkinson's disease: clinical trial results from a randomized, placebo-controlled design. Gut Microb.

[145] Bruggeman A., Vandendriessche C., Hamerlinck H., De Looze D., Tate D.J., Vuylsteke M. (2024 May 1). Safety and efficacy of faecal microbiota transplantation in patients with mild to moderate Parkinson's disease (GUT-PARFECT): a double-blind, placebo-controlled, randomised, phase 2 trial. EClinicalMedicine.

[146] Scheperjans F., Levo R., Bosch B., Lääperi M., Pereira P.A.B., Smolander O.P. (2024). Fecal microbiota transplantation for treatment of Parkinson disease: a randomized clinical trial. JAMA Neurol.

[147] Walsh S., Wallace L., Kuhn I., Mytton O., Lafortune L., Wills W. (2024 Apr 1). Population-level interventions for the primary prevention of dementia: a complex evidence review. eClinicalMedicine.

[148] Yan P., Kim K.W., Xiao Q., Ma X., Czerniewski L.R., Liu H. (2022 Jun 1). Peripheral monocyte-derived cells counter amyloid plaque pathogenesis in a mouse model of Alzheimer's disease. J Clin Invest.

[149] MacPherson K.P., Eidson L.N., Houser M.C., Weiss B.E., Gollihue J.L., Herrick M.K. (2023). Soluble TNF mediates amyloid-independent, diet-induced alterations to immune and neuronal functions in an Alzheimer's disease mouse model. Front Cell Neurosci.

[150] MacPherson K.P., Sompol P., Kannarkat G.T., Chang J., Sniffen L., Wildner M.E. (2017 Jun 1). Peripheral administration of the soluble TNF inhibitor XPro1595 modifies brain immune cell profiles, decreases beta-amyloid plaque load, and rescues impaired long-term potentiation in 5xFAD mice. Neurobiol Dis.

[151] Monoranu C.M., Hartmann T., Strobel S., Heinsen H., Riederer P., Distel L. (2021). Is there any evidence of monocytes involvement in Alzheimer's disease? A pilot study on human postmortem brain. J Alzheimer’s Dis reports..

[152] Muñoz-Castro C., Mejias-Ortega M., Sanchez-Mejias E., Navarro V., Trujillo-Estrada L., Jimenez S. (2023 Dec 1). Monocyte-derived cells invade brain parenchyma and amyloid plaques in human Alzheimer's disease hippocampus. Acta Neuropathol Commun.

[153] Fernández Zapata C., Giacomello G., Spruth E.J., Middeldorp J., Gallaccio G., Dehlinger A. (2022 Dec 1). Differential compartmentalization of myeloid cell phenotypes and responses towards the CNS in Alzheimer's disease. Nat Commun.

[154] Noz M.P., Ter Telgte A., Wiegertjes K., Joosten L.A.B., Netea M.G., De Leeuw F.E. (2018). Trained immunity characteristics are associated with progressive cerebral small vessel disease. Stroke.

[155] Tsai A.S., Berry K., Beneyto M.M., Gaudilliere D., Ganio E.A., Culos A. (2019 Apr 1). A year-long immune profile of the systemic response in acute stroke survivors. Brain.

[156] García-Culebras A., Cuartero M.I., Peña-Martínez C., Moraga A., Vázquez-Reyes S., de Castro-Millán F.J. (2024 Mar 1). Myeloid cells in vascular dementia and Alzheimer's disease: possible therapeutic targets?. Br J Pharmacol.

[157] Zou X., Zou G., Zou X., Wang K., Chen Z. (2024 Mar 13). Gut microbiota and its metabolites in Alzheimer's disease: from pathogenesis to treatment. PeerJ.

[158] Ji D., Chen W.Z., Zhang L., Zhang Z.H., Chen L.J. (2024 Dec 1). Gut microbiota, circulating cytokines and dementia: a Mendelian randomization study. J Neuroinflammation.

[159] Scarmeas N., Stern Y., Tang M.X., Mayeux R., Luchsinger J.A. (2006 Jun 1). Mediterranean diet and risk for Alzheimer's disease. Ann Neurol.

[160] Olsson E., Karlström B., Kilander L., Byberg L., Cederholm T., Sjögren P. (2015 Jan 1). Dietary patterns and cognitive dysfunction in a 12-year follow-up study of 70 Year old men. J Alzheim Dis.

[161] Xiao J., Katsumata N., Bernier F., Ohno K., Yamauchi Y., Odamaki T. (2020). Probiotic Bifidobacterium breve in improving cognitive functions of older adults with suspected mild cognitive impairment: a randomized, double-blind, placebo-controlled trial. J Alzheimers Dis.

[162] Kobayashi Y., Kuhara T., Oki M., Xiao J.Z. (2019). Effects of Bifidobacterium breve A1 on the cognitive function of older adults with memory complaints: a randomised, double-blind, placebo-controlled trial. Benef Microbes.

[163] Hwang Y.H., Park S., Paik J.W., Chae S.W., Kim D.H., Jeong D.G. (2019). Efficacy and safety of Lactobacillus plantarum C29-fermented soybean (DW2009) in individuals with mild cognitive impairment: a 12-week, multi-center, randomized, double-blind, placebo-controlled clinical trial. Nutrients.

[164] Akbari E., Asemi Z., Kakhaki R.D., Bahmani F., Kouchaki E., Tamtaji O.R. (2016 Nov 10). Effect of probiotic supplementation on cognitive function and metabolic status in Alzheimer's disease: a randomized, double-blind and controlled trial. Front Aging Neurosci.

[165] Akhgarjand C., Vahabi Z., Shab-Bidar S., Etesam F., Djafarian K. (2022 Oct 31). Effects of probiotic supplements on cognition, anxiety, and physical activity in subjects with mild and moderate Alzheimer's disease: a randomized, double-blind, and placebo-controlled study. Front Aging Neurosci.

[166] Fei Y., Wang R., Lu J., Peng S., Yang S., Wang Y. (2023 May 1). Probiotic intervention benefits multiple neural behaviors in older adults with mild cognitive impairment. Geriatr Nurs.

[167] Sakurai K., Toshimitsu T., Okada E., Anzai S., Shiraishi I., Inamura N. (2022 Oct 1). Effects of lactiplantibacillus plantarum OLL2712 on memory function in older adults with declining memory: a randomized placebo-controlled trial. Nutrients.

[168] Asaoka D., Xiao J., Takeda T., Yanagisawa N., Yamazaki T., Matsubara Y. (2022). Effect of probiotic Bifidobacterium breve in improving cognitive function and preventing brain atrophy in older patients with suspected mild cognitive impairment: results of a 24-week randomized, double-blind, placebo-controlled trial. J Alzheimers Dis.

[169] Hsu Y.C., Huang Y.Y., Tsai S.Y., Kuo Y.W., Lin J.H., Ho H.H. (2023 Jan 1). Efficacy of probiotic supplements on brain-derived neurotrophic factor, inflammatory biomarkers, oxidative stress and cognitive function in patients with Alzheimer's dementia: a 12-week randomized, double-blind active-controlled study. Nutrients.

[170] Nassar S.T., Tasha T., Desai A., Bajgain A., Ali A., Dutta C. (2022 Oct 6). Fecal microbiota transplantation role in the treatment of Alzheimer's disease: a systematic review. Cureus.

[171] Hazan S. (2020 Jun 30). Rapid improvement in Alzheimer's disease symptoms following fecal microbiota transplantation: a case report.

[172] Park S.H., Lee J.H., Shin J., Kim J.S., Cha B., Lee S. (2021). Cognitive function improvement after fecal microbiota transplantation in Alzheimer's dementia patient: a case report. Curr Med Res Opin.

[173] Park S.H., Lee J.H., Kim J.S., Kim T.J., Shin J., Im J.H. (2022 Aug 8). Fecal microbiota transplantation can improve cognition in patients with cognitive decline and Clostridioides difficile infection. Aging (Albany NY).

[174] Chen X., Zhang W., Lin Z., Zheng C., Chen S., Zhou H. (2023). Preliminary evidence for developing safe and efficient fecal microbiota transplantation as potential treatment for aged related cognitive impairments. Front Cell Infect Microbiol.

[175] Fani Maleki A., Rivest S. (2019 Jul 31). Innate immune cells: monocytes, monocyte-derived macrophages and microglia as therapeutic targets for Alzheimer's disease and multiple sclerosis. Front Cell Neurosci.

[176] Maglione A., Zuccalà M., Tosi M., Clerico M., Rolla S. (2021 Jul 29). Host genetics and gut microbiome: perspectives for multiple sclerosis. Genes.

[177] Chen J., Chia N., Kalari K.R., Yao J.Z., Novotna M., Soldan M.M.P. (2016 Jun 27). Multiple sclerosis patients have a distinct gut microbiota compared to healthy controls. Sci Rep.

[178] Miyake S., Kim S., Suda W., Oshima K., Nakamura M., Matsuoka T. (2015 Sep 14). Dysbiosis in the gut microbiota of patients with multiple sclerosis, with a striking depletion of species belonging to clostridia XIVa and IV clusters. PLoS One.

[179] Berer K., Gerdes L.A., Cekanaviciute E., Jia X., Xiao L., Xia Z. (2017 Oct 3). Gut microbiota from multiple sclerosis patients enables spontaneous autoimmune encephalomyelitis in mice. Proc Natl Acad Sci USA.

[180] Jangi S., Gandhi R., Cox L.M., Li N., Von Glehn F., Yan R. (2016 Jun 28). Alterations of the human gut microbiome in multiple sclerosis. Nat Commun.

[181] Mirza A., Mao-Draayer Y. (2017 Oct 1). The gut microbiome and microbial translocation in multiple sclerosis. Clin Immunol.

[182] Ntranos A., Park H.J., Wentling M., Tolstikov V., Amatruda M., Inbar B. (2022 Feb 1). Bacterial neurotoxic metabolites in multiple sclerosis cerebrospinal fluid and plasma. Brain.

[183] Caravagna C., Jaouën A., Desplat-Jégo S., Fenrich K.K., Bergot E., Luche H. (2018 Dec 1). Diversity of innate immune cell subsets across spatial and temporal scales in an EAE mouse model. Sci Rep.

[184] Johanson D.M., Goertz J.E., Marin I.A., Costello J., Overall C.C., Gaultier A. (2020 Dec 1). Experimental autoimmune encephalomyelitis is associated with changes of the microbiota composition in the gastrointestinal tract. Sci Rep.

[185] Duc D., Vigne S., Bernier-Latmani J., Yersin Y., Ruiz F., Gaïa N. (2019 Oct 8). Disrupting myelin-specific Th17 cell gut homing confers protection in an adoptive transfer experimental autoimmune encephalomyelitis. Cell Rep.

[186] Bauer I.J., Fang P., Lämmle K.F., Tyystjärvi S., Alterauge D., Baumjohann D. (2023). Visualizing the activation of encephalitogenic T cells in the ileal lamina propria by in vivo two-photon imaging. Proc Natl Acad Sci USA.

[187] Monaghan K.L., Zheng W., Hu G., Wan E.C.K. (2019 Nov 26). Monocytes and monocyte-derived antigen-presenting cells have distinct gene signatures in experimental model of multiple sclerosis. Front Immunol.

[188] Moravejolahkami A.R., Chitsaz A., Hassanzadeh A., Paknahad Z. (2023). Effects of anti-Inflammatory-antioxidant-rich diet and co-supplemented synbiotics intervention in patients with progressive forms of multiple sclerosis: a single-center, single-blind randomized clinical trial. Nutr Neurosci.

[189] Harirchian M.H., Karimi E., Bitarafan S. (2022 Jan 1). Diet and disease-related outcomes in multiple sclerosis: a systematic review of clinical trials. Curr J Neurol.

[190] Yadav V., Marracci G., Kim E., Spain R., Cameron M., Overs S. (2016 Sep 1). Low-fat, plant-based diet in multiple sclerosis: a randomized controlled trial. Mult Scler Relat Disord.

[191] Saresella M., Mendozzi L., Rossi V., Mazzali F., Piancone F., LaRosa F. (2017 Oct 25). Immunological and clinical effect of diet modulation of the gut microbiome in multiple sclerosis patients: a pilot study. Front Immunol.

[192] Choi I.Y., Piccio L., Childress P., Bollman B., Ghosh A., Brandhorst S. (2016 Jun 7). A diet mimicking fasting promotes regeneration and reduces autoimmunity and multiple sclerosis symptoms. Cell Rep.

[193] Weinstock-Guttman B., Baier M., Park Y., Feichter J., Lee-Kwen P., Gallagher E. (2005 Nov). Low fat dietary intervention with omega-3 fatty acid supplementation in multiple sclerosis patients. Prostaglandins Leukot Essent Fatty Acids.

[194] Riccio P., Rossano R., Larocca M., Trotta V., Mennella I., Vitaglione P. (2016 Mar 1). Anti-inflammatory nutritional intervention in patients with relapsing-remitting and primary-progressive multiple sclerosis: a pilot study. Exp Biol Med.

[195] Irish A.K., Erickson C.M., Wahls T.L., Snetselaar L.G., Darling W.G. (2017 Jan). Randomized control trial evaluation of a modified Paleolithic dietary intervention in the treatment of relapsing-remitting multiple sclerosis: a pilot study. Degener Neurol Neuromuscul Dis.

[196] Ramirez-Ramirez V., Macias-Islas M.A., Ortiz G.G., Pacheco-Moises F., Torres-Sanchez E.D., Sorto-Gomez T.E. (2013). Efficacy of fish oil on serum of TNF α , IL-1 β , and IL-6 oxidative stress markers in multiple sclerosis treated with interferon beta-1b. Oxid Med Cell Longev.

[197] Torres-Sánchez E.D., Pacheco-Moisés F.P., Macias-Islas M.A., Morales-Sánchez E.W., Ramírez-Ramírez V., Celis De La Rosa A.J. (2018). Effect of fish and olive oil on mitochondrial ATPase activity and membrane fluidity in patients with relapsing-remitting multiple sclerosis treated with interferon beta 1-b. Nutr Hosp.

[198] Zandi-Esfahan S., Fazeli M., Shaygannejad V., Hasheminia J., Badihian S., Aghayerashti M. (2017 Dec 1). Evaluating the effect of adding Fish oil to Fingolimod on TNF-α, IL1β, IL6, and IFN-γ in patients with relapsing-remitting multiple sclerosis: a double-blind randomized placebo-controlled trial. Clin Neurol Neurosurg.

[199] Rezapour-Firouzi S., Arefhosseini S.R., Mehdi F., Mehrangiz E.M., Baradaran B., Sadeghihokmabad E. (2013 Oct). Immunomodulatory and therapeutic effects of Hot-nature diet and co-supplemented hemp seed, evening primrose oils intervention in multiple sclerosis patients. Compl Ther Med.

[200] Majdinasab N., Namjoyan F., Taghizadeh M., Saki H. (2018 Jun 12). The effect of evening primrose oil on fatigue and quality of life in patients with multiple sclerosis. Neuropsychiatric Dis Treat.

[201] Adalat M., Khalili M., Ayromlou H., Haririan S., Rezaeizadeh H., Safari A.A. (2018). Anti-fatigue and hypnotic effects of a traditional herbal extract on multiple sclerosis patients : a double blind randomized clinical trial. Milddle East J Fam Med.

[202] Coe S., Cossington J., Collett J., Soundy A., Izadi H., Ovington M. (2019 May 1). A randomised double-blind placebo-controlled feasibility trial of flavonoid-rich cocoa for fatigue in people with relapsing and remitting multiple sclerosis. J Neurol Neurosurg Psychiatry.

[203] Coe S., Axelsson E., Murphy V., Santos M., Collett J., Clegg M. (2017 Oct 1). Flavonoid rich dark cocoa may improve fatigue in people with multiple sclerosis, yet has no effect on glycaemic response: an exploratory trial. Clin Nutr ESPEN.

[204] Fleming J.O., Isaak A., Lee J.E., Luzzio C.C., Carrithers M.D., Cook T.D. (2011). Probiotic helminth administration in relapsing-remitting multiple sclerosis: a phase 1 study. Mult Scler.

[205] Tanasescu R., Tench C.R., Constantinescu C.S., Telford G., Singh S., Frakich N. (2020 Sep 1). Hookworm treatment for relapsing multiple sclerosis: a randomized double-blinded placebo-controlled trial. JAMA Neurol.

[206] Tamtaji O.R., Kouchaki E., Salami M., Aghadavod E., Akbari E., Tajabadi-Ebrahimi M. (2017 Nov 17). The effects of probiotic supplementation on gene expression related to inflammation, insulin, and lipids in patients with multiple sclerosis: a randomized, double-blind, placebo-controlled trial. J Am Coll Nutr.

[207] Kouchaki E., Tamtaji O.R., Salami M., Bahmani F., Daneshvar Kakhaki R., Akbari E. (2017 Oct 1). Clinical and metabolic response to probiotic supplementation in patients with multiple sclerosis: a randomized, double-blind, placebo-controlled trial. Clin Nutr.

[208] Salami M., Kouchaki E., Asemi Z., Tamtaji R. (2018). How probiotic bacteria influence the motor and mental behaviors as well as immunological and oxidative biomarkers in multiple sclerosis? A double blind clinical trial. J Funct Foods.

[209] Asghari K.M., Dolatkhah N., Ayromlou H., Mirnasiri F., Dadfar T., Hashemian M. (2023 Dec 1). The effect of probiotic supplementation on the clinical and para-clinical findings of multiple sclerosis: a randomized clinical trial. Sci Rep.

[210] Tankou S.K., Regev K., Healy B.C., Tjon E., Laghi L., Cox L.M. (2018 Jun 1). A probiotic modulates the microbiome and immunity in multiple sclerosis. Ann Neurol.

[211] Makkawi S., Camara-Lemarroy C., Metz L. (2018 Jul 1). Fecal microbiota transplantation associated with 10 years of stability in a patient with SPMS. Neurol Neuroimmunol NeuroInflammation.

[212] Garcia-Rodriguez V., Ali S.I., Dupont A.W. (2020 Oct). S2314 fecal microbiota transplantation associated with disease stabilization in a patient with multiple sclerosis. Am J Gastroenterol.

[213] Engen P.A., Zaferiou A., Rasmussen H., Naqib A., Green S.J., Fogg L.F. (2020 Sep 8). Single-arm, non-randomized, time series, single-subject study of fecal microbiota transplantation in multiple sclerosis. Front Neurol.

[214] Al K.F., Craven L.J., Gibbons S., Parvathy S.N., Wing A.C., Graf C. (2022 Apr 1). Fecal microbiota transplantation is safe and tolerable in patients with multiple sclerosis: a pilot randomized controlled trial. Mult Scler J - Exp Transl Clin.

